# Conditional teleportation of quantum-dot spin states

**DOI:** 10.1038/s41467-020-16745-0

**Published:** 2020-06-15

**Authors:** Haifeng Qiao, Yadav P. Kandel, Sreenath K. Manikandan, Andrew N. Jordan, Saeed Fallahi, Geoffrey C. Gardner, Michael J. Manfra, John M. Nichol

**Affiliations:** 1 Department of Physics and Astronomy, University of Rochester, Rochester, NY 14627 USA; 2 Institute for Quantum Studies, Chapman University, Orange, CA 92866 USA; 3 Department of Physics and Astronomy, Purdue University, West Lafayette, IN 47907 USA; 4 Birck Nanotechnology Center, Purdue University, West Lafayette, IN 47907 USA; 5 School of Materials Engineering, Purdue University, West Lafayette, IN 47907 USA; 6 School of Electrical and Computer Engineering, Purdue University, West Lafayette, IN 47907 USA

**Keywords:** Quantum dots, Quantum information

## Abstract

Among the different platforms for quantum information processing, individual electron spins in semiconductor quantum dots stand out for their long coherence times and potential for scalable fabrication. The past years have witnessed substantial progress in the capabilities of spin qubits. However, coupling between distant electron spins, which is required for quantum error correction, presents a challenge, and this goal remains the focus of intense research. Quantum teleportation is a canonical method to transmit qubit states, but it has not been implemented in quantum-dot spin qubits. Here, we present evidence for quantum teleportation of electron spin qubits in semiconductor quantum dots. Although we have not performed quantum state tomography to definitively assess the teleportation fidelity, our data are consistent with conditional teleportation of spin eigenstates, entanglement swapping, and gate teleportation. Such evidence for all-matter spin-state teleportation underscores the capabilities of exchange-coupled spin qubits for quantum-information transfer.

## Abstract (web summary)

Despite recent demonstrations of coherent spin-state transfer in arrays of spin qubits via exchange interaction, all-matter spin-state teleportation is still out of reach. Here the authors provide evidence for conditional teleportation of quantum-dot spin states, entanglement swapping, and gate teleportation.

## Introduction

Quantum teleportation^1^ is an exquisite example of the power of quantum information transfer. Teleportation has been demonstrated in many experimental quantum information processing platforms^2–7^, and it is an essential tool for quantum error correction^8^, measurement-based quantum computing^9^, and quantum gate teleportation^10^. However, quantum teleportation has not previously been demonstrated in quantum-dot spin qubits. Separating entangled pairs of spins to remote locations, as required for quantum teleportation, has previously presented the main challenge to teleportation in quantum dots.

Here, we overcome this challenge using a recently demonstrated technique to distribute entangled spin states via Heisenberg exchange^11^. This technique does not involve the motion of electrons, greatly simplifying the teleportation procedure. Our teleportation method also leverages Pauli spin blockade, a unique feature of electrons in quantum dots, to generate and measure entangled pairs of spins. We combine these concepts to perform conditional teleportation in a system of four GaAs quantum-dot spin qubits. Our data are consistent with conditional teleportation of quantum-dot spin states, entanglement swapping, and gate teleportation. Entanglement swapping^12^ goes beyond teleportation of single-qubit states to create entanglement between uncorrelated particles via measurements, and demonstrations of entanglement swapping in matter qubits are rare^13,14^. Our technique is fully compatible with all gate-defined quantum-dot types, including Si quantum dots. Although we use coherent spin-state transfer via Heinseberg exchange^11^ to distribute entangled pairs of spins, other methods to create long-range-entangled states of spins including tunneling^15–17^ and coupling via superconducting resonators^18^ could be used as well.

## Results

### Device description

**Fig. 1:**
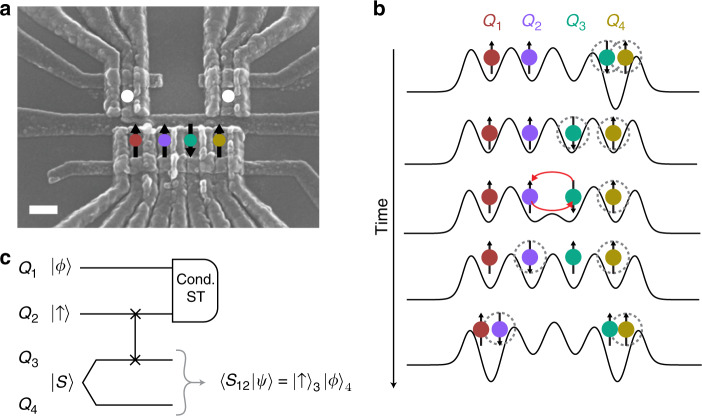
Experimental setup. **a** Scanning electron micrograph of the quadruple quantum dot. The positions of the electron-spin qubits are overlaid. The white dots indicate the positions of the sensor quantum dots. The scale bar is 200 nm. **b** Physical implementation of the teleportation protocol. Dots 3 and 4 are initialized in the singlet configuration via electron exchange with the reservoirs and then separated via tunneling. We implement the SWAP gate as a positive voltage pulse to the barrier gate between dots 2 and 3. Pairs of qubits are measured in the singlet/triplet basis via Pauli spin blockade. **c** Circuit diagram for the conditional quantum teleportation protocol. $\left|\psi \right\rangle$ represents the four-qubit wavefunction.

We implement our teleportation method in a four-qubit quantum processor, which consists of a quadruple quantum dot fabricated in a GaAs/AlGaAs heterostructure [Fig. 1a]. Because the ground state wavefunction of two electrons has the spin-singlet configuration, initialization of two spins in a single quantum dot automatically generates an entangled pair of spins^19,20^. Furthermore, spin-to-charge conversion via Pauli spin blockade^19,21^ enables rapid single-shot measurement of pairs of electron spins in the $\{\left|S\right\rangle ,\left|T\right\rangle \}$ basis, where $\left|T\right\rangle$ is any one of the triplet states $\{\left|\uparrow \uparrow \right\rangle ,\frac{1}{\sqrt{2}}\left(\left|\uparrow \downarrow \right\rangle +\left|\downarrow \uparrow \right\rangle \right),\left|\downarrow \downarrow \right\rangle \}$. We therefore configure the quadruple quantum dot as two pairs of spins to facilitate teleportation. Spins 1 and 2 form the left pair, and spins 3 and 4 form the right pair.

We achieve separation and distribution of entangled pairs of spins through coherent spin-state transfer based on Heisenberg exchange^11^. To transfer a spin state from one electron to another, we induce exchange coupling between electrons by applying a voltage pulse to the barrier gate between them [Fig. 1b]^22,23^. Because exchange coupling generates a SWAP operation, this procedure interchanges the two states. This procedure can be repeated for different pairs of spins to enable long-distance spin-state transfer. Importantly, exchange-based spin swaps preserve entangled states^11^.

### Conditional teleportation protocol

 Figure 1c shows the quantum circuit for our procedure, which can conditionally teleport an arbitrary state $\left|\phi \right\rangle$ from dot 1 to dot 4. We prepare qubit 2 in the $\left|\uparrow \right\rangle$ state, and it is used later for readout, as discussed further below. We generate the Einstein-Podolsky-Rosen (EPR) pair between qubits 3 and 4 by loading two electrons into the right-most dot via electrical exchange with reservoirs. We then separate the two electrons via tunneling. After a SWAP gate on qubits 2 and 3, the EPR pair resides in qubits 2 and 4. To teleport $\left|\phi \right\rangle$ from qubit 1 to qubit 4, we project the left pair of qubits onto the $\{\left|S\right\rangle ,\left|T\right\rangle \}$ basis via diabatic charge transfer into the outer dots^11^ [Fig. 1b]. Our measurements in the $\{\left|S\right\rangle ,\left|T\right\rangle \}$ basis can only distinguish $\left|S\right\rangle =\left|{\Psi }^{-}\right\rangle$ from the other Bell states $\left|{\Psi }^{+}\right\rangle$, $\left|{\Phi }^{+}\right\rangle$, or $\left|{\Phi }^{-}\right\rangle$, which are linear combinations of the triplet states. In this case, therefore, successful teleportation requires obtaining a singlet in the left pair. To verify teleportation, we also project the right pair, using either diabatic or adiabatic charge transfer (see “Methods”).

The utility of quantum teleportation lies in its ability to transmit unknown quantum states. Usually, teleportation of unknown states is experimentally demonstrated by verifying teleportation of a complete set of single-qubit basis states^2^ or through process tomography^5^. Because our four-qubit device does not incorporate a micromagnet or antenna for magnetic resonance, we are not able to prepare superposition states of single spins. Therefore, to illustrate the operation of the teleportation procedure, we first teleport a classical spin state from qubit 1 to qubit 4. Later, we demonstrate entanglement swapping in our four-qubit processor, which conclusively demonstrates non-local manipulation of quantum states via measurements. In the future, quantum state tomography will be required to establish that the teleportation fidelity exceeds the classical bound, as discussed below.

**Fig. 2:**
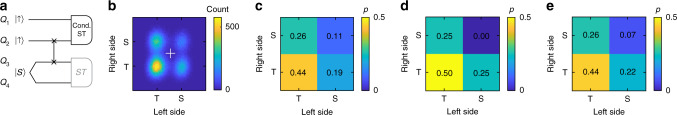
Conditional teleportation of a classical spin state. **a** Quantum circuit to teleport a state $\left|\uparrow \right\rangle$ from qubit 1 to qubit 4. The conditional singlet–triplet measurement on qubits 1 and 2 induces teleportation, and the gray singlet–triplet measurement of the right pair verifies teleportation. **b** Experimentally measured probability distribution for 65,536 single-shot realizations of the teleportation sequence in **a**. The white cross indicates the threshold used to calculate probabilities. **c** Extracted probabilities *p* from the distribution in **b**. **d** Simulated probabilities computed neglecting any errors. **e** Simulated probabilities accounting for readout errors, state preparation errors, charge noise, and hyperfine fields. All probabilities are rounded to the nearest hundredth.

To demonstrate the basic operation of our teleportation method using $\left|\phi \right\rangle =\left|\uparrow \right\rangle$, we prepare qubits 3 and 4 in a spin singlet [Fig. 2a]. Qubits 1 and 2 are prepared in the $\left|{\psi }_{12}\right\rangle ={\left|\phi \right\rangle }_{1}{\left|\uparrow \right\rangle }_{2}={\left|\uparrow \right\rangle }_{1}{\left|\uparrow \right\rangle }_{2}$ state by electrical exchange with the reservoirs (see “Methods”). After the SWAP operation, if the left pair projects onto $\left|{S}_{12}\right\rangle$, qubit 4 should be identically $\left|\uparrow \right\rangle$^1^. Because qubit 3 has the $\left|\uparrow \right\rangle$ state (a result of the earlier SWAP operation), the right pair should be in the $\left|{\psi }_{34}\right\rangle ={\left|\uparrow \right\rangle }_{3}{\left|\phi \right\rangle }_{4}={\left|\uparrow \right\rangle }_{3}{\left|\uparrow \right\rangle }_{4}$ state, and measuring a singlet on the left pair should perfectly correlate with measuring a triplet on the right pair. Figure 2b displays a joint histogram of 65, 536 single-shot measurements on both pairs of qubits for the teleport operation discussed above. Figure 2c shows the extracted probabilities for the different outcomes. Our measurements closely match the predicted probabilities, as shown in Fig. 2d (see “Methods”). Figure 2e shows a prediction including known sources of experimental error, including readout fidelity, relaxation during readout, state preparation error, charge noise, and hyperfine fields, and this prediction matches the observed data closely. We discuss these errors further below. We have also performed similar experiments with qubit 1 prepared in a mixed state (Supplementary Fig. 1), and the results are consistent with our expectations.

**Fig. 3:**
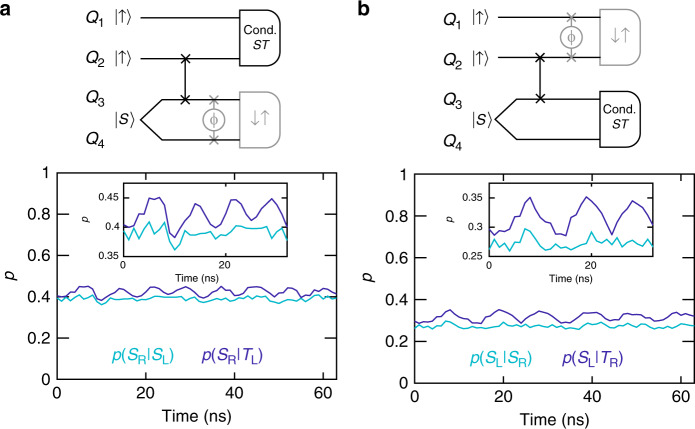
Verification of conditional teleportation of a classical state. **a** We apply an exchange pulse to qubits 3 and 4 after measuring qubits 1 and 2. Here, *ϕ* = 2*πJ*_34_*t*, where *J*_34_ is the induced exchange coupling between qubits 3 and 4, and *t* is the evolution time given by the *x*-coordinate of each data point. (*ϕ* = *π* corresponds to a SWAP operation.) When the left pair give a singlet, the right pair have the same spin, and no oscillations should be visible. The inset shows the same data from 0 to 32 ns. **b** Applying an exchange pulse to the left pair after measuring the right pair generates exchange oscillations on the left pair only if the right pair yields a triplet. The inset shows the same data from 0 to 32 ns. Here, *ϕ* = 2*πJ*_12_*t*, where *J*_12_ is the induced exchange coupling between qubits 1 and 2. In both panels, each data point represents the average of 16, 384 single-shot measurements, and the gray elements of the circuits serve to verify teleportation.

To verify conditional teleportation of the classical state, we perform an exchange gate on qubits 3 and 4 following the teleport [Fig. 3a]. In the case of successful teleportation, qubits 3 and 4 should have the $\left|{\psi }_{34}\right\rangle ={\left|\uparrow \right\rangle }_{3}{\left|\uparrow \right\rangle }_{4}$ state, and the exchange gate should have no effect. Indeed, after measuring a singlet on the left pair, we do not observe significant exchange oscillations on the right pair, but after measuring a triplet on the left pair, we do observe exchange oscillations on the right pair [Fig. 3a]. As shown in Supplementary Fig. 2, eliminating the SWAP operation between qubits 2 and 3 or preparing a product state, instead of an EPR pair, on the right side, largely eliminate the conditional effect, consistent with our simulations (Supplementary Fig. 3). These data demonstrate that both the EPR pair and the SWAP operation are critical for teleportation, as expected.

The circuit of Fig. 2a can also teleport the state of qubit 3 to qubit 2, depending on the result of the right-pair measurement. To verify that teleportation can also occur from qubit 3 to qubit 2, we switched the order of measurements and performed the variable exchange gate on the left pair of qubits [Fig. 3b]. In this case, we observe that the oscillations on the left pair depend on the state of the right pair. Again, removing the SWAP operation or the EPR pair significantly eliminates the conditional effect (Supplementary Fig. 2). We have performed simulations (see “Methods”), which include known sources of error, that match our observed data closely, as shown in Supplementary Fig. 3. Our simulations reproduce the weak residual oscillations in *p*(*S*_L_∣*S*_R_) and *p*(*S*_R_∣*S*_L_) (Fig. 3), which likely result from an imperfect SWAP operation and readout errors. Supplementary Fig. 4 shows the expected ideal results for these measurements in the absence of any errors.

### Conditional entanglement swapping and gate teleportation

**Fig. 4:**
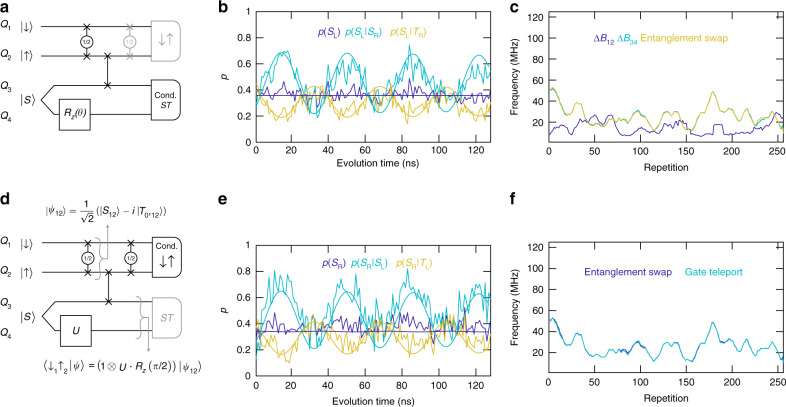
Conditional entanglement swapping and gate teleportation. **a** Circuit diagram for conditional entanglement swapping. The gray circuit elements are used to verify entanglement swapping. **b** The unconditioned singlet probability on the left side *p*(*S*_L_) shows no oscillations. However, *p*(*S*_L_∣*S*_R_) and *p*(*S*_L_∣*T*_R_) show pronounced singlet–triplet oscillations. Simulated predictions are shown in the same color. **c** Comparison of the extracted oscillation frequency vs. repetition number measured on qubits 1 and 2 to the measured hyperfine gradients. The sudden jump in Δ*B*_12_ near repetition 200 likely results from frequencies too low to measure properly. **d** Circuit diagram for conditional gate teleportation. **e** *p*(*S*_R_) shows only weak oscillations, but *p*(*S*_R_∣*S*_L_) and *p*(*S*_R_∣*T*_L_) show prominent singlet–triplet oscillations. Simulated predictions are shown in the same color. **f** Comparison of the extracted entanglement-swap and gate-teleport oscillation frequencies vs. repetition number.

Having illustrated the basic operation of the teleport procedure, we now present evidence for conditional entanglement swapping, which confirms that the four-qubit processor indeed performs non-local coherent manipulation of quantum information using measurements [Fig. 4a]. Entanglement swapping^12^ uses teleportation to generate entanglement between uncorrelated particles via measurements. In this case, we prepare the EPR state between qubits 1 and 2 via a $\sqrt{\,\text{SWAP}\,}$ gate, starting from the ${\left|\downarrow \right\rangle }_{1}{\left|\uparrow \right\rangle }_{2}$ state. This process generates the entangled state $\frac{1}{\sqrt{2}}\left(\left|{S}_{12}\right\rangle -i\left|{T}_{0,12}\right\rangle \right)$, where $\left|{T}_{0}\right\rangle =\frac{1}{\sqrt{2}}\left(\left|\uparrow \downarrow \right\rangle +\left|\downarrow \uparrow \right\rangle \right)$. At the same time, we prepare a separated singlet between qubits 3 and 4. Before teleportation, we evolve the separated singlet in its local hyperfine gradient Δ*B*_34_ for a variable time *t*. This evolution generates an effective *z*-rotation on qubit 4 relative to qubit 3 by an angle *θ* = *gμ*_B_Δ*B*_34_*t*/*ℏ*, where *g* is the electron *g* factor in GaAs, and *μ*_B_ is the Bohr magneton. The *z*-rotation on qubit 4 coherently rotates the joint state of qubits 3 and 4 to $\cos (\theta /2+\pi /4)\left|S\right\rangle +\exp (-i\pi /2)\sin (\theta /2+\pi /4)\left|{T}_{0}\right\rangle$^19,20^. During this evolution time, qubits 3 and 4 remain maximally entangled.

After a SWAP gate between qubits 2 and 3, qubits 1 and 3 are entangled, and qubits 2 and 4 are entangled. After projection to a singlet on the right side, entanglements have been swapped, because the entangled state of qubit 4 is teleported to qubit 1. Qubits 1 and 2, which were not entangled immediately before the measurement, become entangled, provided qubits 3 and 4 project onto the singlet state. Moreover, the coherent singlet–triplet evolution that occurred on qubits 3 and 4 should appear on qubits 1 and 2, given a singlet outcome on the right pair (see Supplementary Note 1). To verify entanglement swapping, we measure the left pair of qubits by adiabatic charge transfer^19,20^ (see “Methods”) following another $\sqrt{{\rm{SWAP}}}$ gate. In the case of successful entanglement swapping, the final $\sqrt{{\rm{SWAP}}}$ gate preserves the coherence of the teleported state against the effects of hyperfine fluctuations during readout.

To observe the anticipated oscillations, we sweep *t*, which controls the *z* rotation on qubit 4, from 0 to 127 ns, in steps of 1 ns. For each time interval, we implement the quantum circuit shown in Fig. 4a and record a single-shot measurement of both pairs of qubits, and we average this set of measurements 256 times. Figure 4b shows the average of one such set of measurements. No oscillations are visible in the unconditioned singlet probability of the left pair *p*(*S*_L_). However, prominent oscillations are visible in the probability of a singlet on the left given a singlet on the right *p*(*S*_L_∣*S*_R_) and also in *p*(*S*_L_∣*T*_R_), in good agreement with our simulations [Fig. 4b and Supplementary Fig. 6]. These oscillations demonstrate conditional entanglement swapping.

Because the nuclear hyperfine fields fluctuate in time, we repeat this set of measurements 256 times, and the entire data set is shown in Supplementary Fig. 7. In between each set, we also perform additional measurements to determine the hyperfine gradients between dots 1 and 2 (Δ*B*_12_) and dots 3 and 4 (Δ*B*_34_) (Supplementary Fig. 8)^20^. In total, each repetition takes about one second.

For each repetition, we extract the oscillation frequency by taking a fast Fourier transform of the data (see “Methods” and Supplementary Fig. 7). Figure 4c shows the extracted oscillation frequency that appears on qubits 1 and 2 after entanglement swapping in addition to the frequencies corresponding to Δ*B*_12_ and Δ*B*_34_, which were measured concurrently with the teleportation. The observed oscillation frequency measured on qubits 1 and 2 clearly matches the measured hyperfine gradient Δ*B*_34_. Because Δ*B*_12_ and Δ*B*_34_ result from independent nuclear spin ensembles, they evolve differently in time. We note the good agreement between the time evolution of the oscillation frequency after entanglement swapping and the gradient Δ*B*_34_.

To confirm that the singlet–triplet oscillations on the left pair result from entanglement swapping, we have performed additional measurements which omit the SWAP operation between qubits 2 and 3 (Supplementary Fig. 5). These data show no conditional effect. Therefore, the observed oscillations on qubits 1 and 2 in Fig. 4b result entirely from the coherent evolution between entangled states of qubits 3 and 4, together with the SWAP gate and Bell-state measurement. This demonstration of entanglement swapping using our four-qubit processor confirms that we can perform non-local coherent manipulation on entangled states of the form $\cos (\theta /2)\left|S\right\rangle +\exp ({\!}\pm {\!}i\pi /2)\sin (\theta /2)\left|{T}_{0}\right\rangle$ by quantum measurements.

A similar circuit [Fig. 4d] also implements a simple example of conditional quantum gate teleportation^10^, provided that we post-select on the left-side measurements, instead of the right side. In this case, the EPR pair initially consists of qubits 3 and 4, and we teleport qubit 1 to qubit 4. A unitary gate *U* (the same *z* rotation discussed above), which is applied to one member of the EPR pair before teleportation, appears on qubit 4 after teleportation. The initial entangled state of qubits 1 and 2 is $\left|{\psi }_{12}\right\rangle =\frac{1}{\sqrt{2}}\left(\left|{S}_{12}\right\rangle -i\left|{T}_{0,12}\right\rangle \right)$. Following the SWAP and conditional teleportation of qubit 1 to qubit 4, qubits 3 and 4 have the state $\left|{\psi }_{34}\right\rangle =({\mathbb{1}}\otimes U\cdot {R}_{z}(\pi /2))\left|{\psi }_{12}\right\rangle$, and *U* has been applied to qubit 4. The added *z* rotation on qubit 4 occurs because of the additional $\sqrt{{\rm{SWAP}}}$ and measurement via adiabatic charge transfer on the left side [Fig. 4d].

We measure the right pair of qubits via diabatic charge transfer to verify teleportation [Fig. 4e]. The unconditioned data show very weak oscillations, likely due to an imperfect SWAP gate^11^. Post-selecting based on singlet outcomes on the left side yields prominent oscillations in time, consistent with our simulations. The extracted oscillation frequency versus repetition number agrees well with the data from Fig. 4c, as shown in Fig. 4f.

The fidelity of the teleport operation is limited by readout fidelity, relaxation during readout, state preparation, charge noise, and the hyperfine coupling between the electron spins and Ga and As nuclear spins in the substrate. Readout fidelity and relaxation both limit the probability that we will correctly measure the Bell state of one of the EPR pair and the qubit to be teleported. Readout fidelities are 0.93 for the left pair and 0.87 for the right pair (see “Methods” and Supplementary Figs. 11 and 12). State preparation of the EPR pair also affects the teleport operation. We estimate the probability that we correctly prepare the singlet state in dots 3–4 is 0.89, based on our experimental characterization of the loading process [Supplementary Fig. 10b]. Charge noise causes dephasing of the SWAP operation, and the nuclear hyperfine field limits the fidelity of the SWAP operation that we use to transmit the entangled pair of electrons^11^. The simulations shown in Figs. 2 and 4 and Supplementary Figs. 1, 3, and 5 include all of these effects, in addition to the classical-state initialization error (see “Methods”) where appropriate.

To assess the fidelity of the teleport operation itself for classical states, we simulated the circuit shown in Fig. 2a, assuming perfect state preparation of the left pair, but including all other sources of error. Based on our simulations, we expect that the spin in dot 4 will be in the $\left|\uparrow \right\rangle$ state after the teleport with a probability of about 0.9, given a singlet on the left pair. In the presence of realistic hyperfine gradients (tens of MHz) and exchange strengths (several hundred MHz), we estimate that readout errors contribute the majority of the error.

Assuming perfect preparation of a separated singlet state, our simulations suggest that the fidelity of the entanglement swap [Fig. 4a] on a singlet state can be ~0.7, provided that the state is allowed to evolve in the presence of a quasi-static magnetic gradient to undo the coherent singlet–triplet evolution incurred during the SWAP operation in the presence of a gradient^11^ (see Supplementary Note 3). In this case, readout errors, state preparation errors of the EPR pair, and errors in the SWAP gate due to the magnetic gradient all contribute to the overall error. The average classical limit for teleporting entangled states of the type we use in this experiment is 2/3^24^ (see Supplementary Note 2). By fitting the data of Fig. 4b (see “Methods”, Supplementary Note 3, and Supplementary Fig. 9), we can also extract a maximum singlet teleportation probability of 0.71 ± 0.04, which compares favorably with the classical limit, although further research involving quantum state tomography is required to provide definitive proof. This value also agrees with our simulated fidelity and indicates that a classical explanation for our data is extremely unlikely (see Supplementary Notes 1 and 4).

## Discussion

This teleportation protocol is fully compatible with all gate-defined quantum-dot types, including Si quantum dots. Indeed, this teleportation protocol will work best with small magnetic gradients, as can be achieved with Si qubits. In large gradients, resonant approaches^25,26^ or dynamically corrected gates^27^ can still generate high-fidelity SWAP operations. State preparation errors can be suppressed by improving the coupling between the quantum dots and the reservoirs, and readout errors can be minimized by optimizing the position of the sensor quantum dots. We discuss the potential application of this technique to Si qubits in “Methods”.

As mentioned above and discussed further in “Methods”, the conditional quantum teleportation protocol we have developed is compatible with arbitrary qubit states. Deterministic quantum teleportation of arbitrary quantum states can also be realized with measurements of each qubit in the computational basis, together with CNOT^28–30^ and single-qubit gates^31^, which will enable complete measurements in the Bell-state basis^32^. Fast spin measurements together with real-time adaptive control^33^ could be used to complete the deterministic state transfer process.

The evidence we have presented for conditional state teleportation, entanglement swapping, and gate teleportation adds time-honored capabilities to the library of quantum information processing techniques available to spin qubits in quantum dots. Our results also highlight the potential of exchange-coupled spin chains for quantum information transfer. We envision that teleportation will be useful for the creation and manipulation of long-range entangled states and for error correction in quantum-dot spin qubits. As spin-based quantum information processors scale up, maintaining high-connectivity between spins will be critical, and quantum teleportation also opens an essential pathway toward achieving this goal. In many ways, spin qubits in quantum dots are an ideal platform for quantum teleportation, because they offer a straightforward means of generating and measuring entangled states of spins. As a result, we expect that quantum teleportation will find significant use in future spin-based quantum information processing efforts.

## Methods

### Device

The four-qubit processor is a quadruple quantum dot fabricated on a GaAs/AlGaAs hetereostructure with a two-dimensional electron gas located 91 nm below the surface. The Si-doped region has vertical width of 14.3 nm, centered 24 nm below the top surface of the wafer. In this region, the dopant density is 3 × 10^18^ cm^−3^. The two-dimensional electron gas density *n* = 1.5 × 10^11^ cm^−2^ and mobility *μ* = 2.5 × 10^6^ cm^2^ V^−1^ s^−1^ were measured at *T* = 4K.

Quantum dot fabrication proceeds as follows. Following ohmic contact fabrication via a standard metal stack and anneal, 10 nm of Al_2_O_3_ was deposited via atomic layer deposition. Three layers of overlapping aluminum gates^34,35^ were defined via electron beam lithography, thermal evaporation, and liftoff. The gate layers are isolated by a thin native oxide layer. The active area of the device is also covered with a grounded top gate. This is likely to screen the effects of disorder imposed by the oxide. Empirically, we find that overlapping gates are essential for the exchange pulses we use in this work. The quadruple dot is cooled in a dilution refrigerator to a base temperature of ~10 mK. An external magnetic field *B* = 0.5 T is applied in the plane of the semiconductor surface perpendicular to the axis connecting the quantum dots. Using virtual gates^36,37^, we tune the device to the single-occupancy regime.

### Initialization

To load the $\left|{T}_{+,12}\right\rangle ={\left|\uparrow \right\rangle }_{1}{\left|\uparrow \right\rangle }_{2}$ state, we exchange electrons with the reservoirs in the (1, 1) charge configuration^20^. Both the magnetic field and temperature limit the fidelity of this process (Supplementary Fig. 10). We simulated the initialization fidelity by calculating the time-dependent populations of all relevant spin-states during the loading procedure. This simulation process is detailed in ref. ^38^. We assumed an electron temperature of 75 mK and a magnetic field of 0.5 T [Supplementary Fig. 10a]. This is broadly consistent with the electron temperatures we have measured in our setup, which range from 50 to 100 mK. Based on these simulations, we estimate that this state preparation fidelity is ~0.7. The simulations presented here take this preparation error into account. In principle, increasing the magnetic field should improve the fidelity of the $\left|{T}_{+}\right\rangle$ loading process. Empirically, however, we did not observe a substantial enhancement with fields up to 1 T, as has previously been observed^38^. We suspect that unintentional dynamic nuclear polarization significantly modifies the magnetic field at the location of each dot.

To load a separated singlet state, we exchange electrons with the reservoirs in the (0,2) charge configuration^20^. We initialize the right pair of electrons in dot 4 as a singlet with 0.89 probability for a load time of 2 μs [Supplementary Fig. 10b]. This could be improved in the future by optimizing the coupling of the electrons to the source and drain reservoirs. Based on simulations of the Landau–Zener tunneling process to separate the electrons, we estimate that separating the singlet state incurs only a few percent error.

We can initialize either pair of electrons as $\left|\downarrow \uparrow \right\rangle$ or $\left|\uparrow \downarrow \right\rangle$ by adiabatically separating a singlet state^20^. The orientation of the two spins in this product state depends on the orientation of the local hyperfine field.

### Exchange

We induce exchange coupling between pairs of qubits by applying a voltage pulse to the barrier between the respective pair of dots^22,23^. Exchange coupling generated in this way is first-order insensitive to charge noise associated with the plunger gates. Barrier-gate pulses are accompanied by compensation pulses on the plunger gates to keep the dot chemical potentials fixed. For the exchange gates used in this work, we used a combination of barrier-^22,23^, and tilt-controlled^19^ exchange. Empirically, we found that using this combination helps us to boost the exchange strength and improves the fidelity of the SWAP operation. All exchange pulses are optimized at the same tuning used to acquire all data in this work with one electron in each dot. We do not observe that pulsing exchange between two spins generates spurious enhanced exchange coupling elsewhere in the array.

### Readout

Diabatic charge transfer into the outer dots projects the spin state of the separated pair onto the $\{\left|S\right\rangle ,\left|T\right\rangle \}$ basis^19,20^. Adiabatic charge transfer into the outer dots maps either $\left|\downarrow \uparrow \right\rangle$ or $\left|\uparrow \downarrow \right\rangle$ to $\left|S\right\rangle$, depending on the sign of the local magnetic gradient, and it maps all other spin states to triplets^19,20^. Here, “diabatic” or “adiabatic” refer to the speed with which the electrons are recombined relative to the size of the hyperfine gradient. We represent readout by diabatic charge transfer with an “ST” in figures, and we represent readout by adiabatic charge transfer with a “↓↑” in figures. When used to verify teleportation, diabatic charge transfer can only verify teleportation when $\left|\phi \right\rangle =\left|\uparrow \right\rangle$. In principle, however, readout by adiabatic charge transfer could be used to measure qubit 4 in its computational basis. If Δ*B*_34_ were such that ${\left|\uparrow \right\rangle }_{3}{\left|\downarrow \right\rangle }_{4}$ were the ground state, adiabatic charge transfer would map ${\left|\uparrow \right\rangle }_{3}{\left|\downarrow \right\rangle }_{4}$ to a singlet, and ${\left|\uparrow \right\rangle }_{3}{\left|\uparrow \right\rangle }_{4}$ to a triplet. Together with tomographic rotation pulses, such a measurement would enable verification of teleportation of arbitrary states.

In addition to conventional spin-blockade readout on both pairs of electrons, we use a shelving mechanism^39^ to enhance the readout visibility. Using the two sensor quantum dots configured for rf-reflectometry (Fig. 1)^21^, we achieve single-shot readout with integration times of 4 μs on the left side and 6 μs on the right side and fidelities of 0.93 and 0.87, respectively. Relaxation times during readout were 65 μs and 48 μs on the left and right sides. Supplementary Fig. 12a, b show the experimentally measured curves demonstrating the relaxation during readout for both pairs of electrons. Supplementary Figure 11a, b show fits to the readout histograms using Eqs. (1) and (2) in ref. ^21^ for each pair of qubits. In all teleportation measurements, both pairs of qubits are measured sequentially in the same single-shot sequence.

To determine the probabilities for the four different possibilities for joint measurements of both pairs, we fit the total measurement histogram for each pair separately. We determine the threshold for each pair by choosing the signal level that maximizes the visibility^21^. We then use these two thresholds to divide the probability distribution into quadrants. The overall probability is normalized, and we calculate the net probability in each quadrant.

To eliminate any state-dependent crosstalk between qubit pairs during readout, we reload the first pair of electrons that we measure as an $\left|S\right\rangle$ before reading out the next pair for the data in Figs. 2 and 3. For the data in Fig. 4, we additionally implemented a voltage ramp to bring each pair of electrons back to the (1,1) idling point immediately after readout. We empirically find that these procedures eliminate crosstalk during readout. The data in Supplementary Fig. 5 demonstrate that there is negligible readout or control crosstalk in our system.

Improvements to readout can be made by repositioning the sensor quantum dots for maximum differential charge sensitivity to achieve readout errors of  <0.01 in integration times of  <1 μs, as has previously been demonstrated in quantum dot spin qubits^33,40^.

### Simulation

Our simulations include errors associated with state preparation, readout fidelity, relaxation during readout, charge noise, and the fluctuating magnetic gradient. We approximate singlet loading error by creating a two-electron state

1 $$\left|\tilde{S}\right\rangle ={s}_{1}\left|S\right\rangle +{s}_{2}\left|{T}_{0}\right\rangle +{s}_{3}\left|{T}_{+}\right\rangle +{s}_{4}\left|{T}_{-}\right\rangle ,$$

where ∣*s*_1_∣^2^ = *f*_s_, and ∣*s*_2_∣^2^ = ∣*s*_3_∣^2^ = ∣*s*_4_∣^2^ = (1 − *f*_s_)/3. Also, $\left|{T}_{-}\right\rangle =\left|\downarrow \downarrow \right\rangle$, and $\left|{T}_{+}\right\rangle =\left|\uparrow \uparrow \right\rangle$. *f*_s_ = 0.89 is the singlet load fidelity. All coefficients are given random phases for each realization of the simulation. To simulate loading error during adiabatic separation of electrons, we set

2 $$\left|\tilde{G}\right\rangle ={s}_{1}\left|\downarrow \uparrow \right\rangle +{s}_{2}\left|\uparrow \downarrow \right\rangle +{s}_{3}\left|{T}_{+}\right\rangle +{s}_{4}\left|{T}_{-}\right\rangle ,$$

where the coefficients are the same as described above. We use the same coefficients, because the singlet initialization error dominates the error in this process. We also allow the orientation of the spins in this state to change between runs of the simulation as the hyperfine gradient changes. We approximate the $\left|{T}_{+}\right\rangle$ loading error by simulating the loading process as described in ref. ^38^. We directly extract the population coefficients of the other three two-electron spin states. We create a state which is a sum of all two-electron spin states:

3 $$\left|\tilde{{T}_{+}}\right\rangle ={t}_{1}\left|S\right\rangle +{t}_{2}\left|{T}_{0}\right\rangle +{t}_{3}\left|{T}_{+}\right\rangle +{t}_{4}\left|{T}_{-}\right\rangle ,$$

where ∣*t*_*i*_∣^2^ is determined as discussed above. We assign random phases to each of the coefficients during each realization of the simulation.

To simulate the spin-eigenstate teleport operation, we set the initial state of the four-qubit system as

4 $$|{\psi }_{i}\rangle =|{\tilde{T}}_{+,12}\rangle \otimes |{\tilde{S}}_{34}\rangle .$$

To simulate the mixed-state and entangled-state teleport operations, we set the initial state as

5 $$\left|{\psi }_{i}\right\rangle =|{\tilde{G}}_{12}\rangle \otimes \left|{\tilde{S}}_{34}\right\rangle .$$

We incorporate charge noise and the hyperfine magnetic field and their effects on the SWAP operation by directly solving the Schrödinger equation for a four spin system. We generated a simulated SWAP operation from the following Hamiltonian:

6 $${H}_{{\rm{S}}}=\frac{h}{4}{J}_{23}({\sigma }_{x,2}\otimes {\sigma }_{x,3}+{\sigma }_{y,2}\otimes {\sigma }_{y,3}+{\sigma }_{z,2}\otimes {\sigma }_{z,3})$$

7 $$+\, \frac{g{\mu }_{{\rm{B}}}}{2}\mathop{\sum }\limits_{k = 1}^{4}{B}_{k}{\sigma }_{z,k}$$

We assume a fixed exchange coupling of *J*_23_ of 250 MHz between spins 2 and 3, and we adjust the time *T*_*S*_ for the SWAP operation to give a *π* pulse. These parameters correspond closely to the actual experiments. To account for charge noise, we allow the value of *J*_23_ to fluctuate by 1% between simulation runs. We arrive at this level of charge noise via the expression $Q=\frac{J}{\sqrt{2}\pi \delta J}$^41^, where $\frac{\delta J}{J}$ is the fractional electrical noise, using the measured quality factor of 21. For the spin-eigenstate simulation, we set the local nuclear magnetic fields *B*_*k*_ of spin *k* to be (−1, 6, −4, 0) MHz $\times \frac{2h}{g{\mu }_{{\rm{B}}}}$ for the qubits. We also include for each qubit the overall background field of 0.5 T. We allow the nuclear field at each site to fluctuate according to a normal distribution with standard deviation of 12 MHz for qubits 1 and 2 and 10 MHz for qubits 3 and 4. The field and fluctuations are adjusted to improve the agreement between the simulations in Supplementary Figs. 2 and 3. We empirically observe that the hyperfine fields fluctuate during the course of a given data-taking run, and they can even switch sign. Because we do not know a-priori what the hyperfine fields will be, it seems reasonable to treat them as fit parameters, especially since the chosen values fall well within the expected range. The overall evolution of the four-qubit system during the SWAP operation is given by the following propagator: ${S}_{23}=\exp \left(\frac{-i{H}_{{\rm{S}}}{T}_{{\rm{S}}}}{\hslash }\right)$.

The voltage pulses in our setup have finite rise times, which cause the four-qubit system to evolve under the magnetic gradient in the absence of exchange. To simulate this effect, we define

8 $${H}_{{\rm{B}}}=\frac{g{\mu }_{{\rm{B}}}}{2}\mathop{\sum }\limits_{k = 1}^{4}{B}_{k}{\sigma }_{z,k}.$$

Under this Hamiltonian, the wavefunction evolves according to the following propagator: ${U}_{{\rm{B}}}=\exp \left(\frac{-i{H}_{{\rm{B}}}{T}_{{\rm{B}}}}{\hslash }\right)$. In the experiment, all pulses are convolved in software with a Gaussian of width 2 ns before delivery to the qubits, so we set *T*_B_ = 2 ns. To simulate the spin-eigenstate teleport experiment, the simulated final state after the teleport operation is thus $\left|\psi \right\rangle ={U}_{{\rm{B}}}{S}_{23}{U}_{{\rm{B}}}\left|{\psi }_{i}\right\rangle$.

For the simulations presented in Supplementary Figs. 1 and 2, we also accounted for imperfections in our pulsing by allowing for the singlet–triplet state vector to rotate slightly during pulses which should ideally be perfectly diabatic. For example, suddenly separating a singlet is usually accompanied by some evolution toward the ground state of the hyperfine field, because the pulse is not perfectly sudden. We account for this by allowing the effective singlet–triplet state vector to rotate by 7° toward the ground state of the hyperfine field during sudden separation of the singlet and by −7° during readout via diabatic charge transfer. This rotation is implemented as a rotation about the *y* axis in the effective *S* − *T*_0_ subspace for each pair of qubits. The *y* axis is defined by the usual *S* − *T*_0_ Hamiltonian: *Jσ*_*z*_ + Δ*Bσ*_*x*_. The rotation angle of 7° was chosen to match an additional control data set in which we adiabatically measured a singlet prepared via diabatic separation. Ideally, this measurement yields a singlet probability of 0.5. In practice, the measured singlet probability is slightly larger than this due to pulse errors, and 7 degrees was chosen to match the observed return probability.

To compute the expected probabilities in Fig. 2d, e, we calculate all pairs of two-qubit correlators: *C*_*α*,*β*_ = 〈*ψ*∣*α* ⊗ *β*〉〈*α* ⊗ *β*∣*ψ*〉, where *α* (qubits 1 and 2) and *β* (qubits 3 and 4) can be any of $\{\left|S\right\rangle ,\left|{T}_{+}\right\rangle ,\left|{T}_{0}\right\rangle ,\left|{T}_{-}\right\rangle \}$. We calculate the probabilities in Fig. 2 as

9 $${P}_{{\rm{SS}}}={C}_{\left|S\right\rangle ,\left|S\right\rangle },$$

10 $${P}_{{\rm{TT}}}=\sum \limits_ {^{\alpha \ne |S\rangle} _{\beta \ne |S\rangle}}{C}_{\alpha ,\beta },$$

11 $${P}_{{\rm{ST}}}=\sum _{\beta \ne \left|S\right\rangle }{C}_{\left|S\right\rangle ,\beta },$$

12 $${P}_{{\rm{TS}}}=\sum _{\alpha \ne \left|S\right\rangle }{C}_{\alpha ,\left|S\right\rangle }.$$

To simulate readout errors, we define the ${g}_{{\rm{L}}({\rm{R}})}=1-\exp (-{t}_{{\rm{m}}}^{{\rm{L}}({\rm{R}})}/{T}_{1}^{{\rm{L}}({\rm{R}})})$ to be the probabilities that the triplet state on the left (right) side will relax to the singlet during readout. Here ${t}_{{\rm{m}}}^{{\rm{L}}({\rm{R}})}$ is the measurement time, and ${T}_{1}^{{\rm{L}}({\rm{R}})}$ is the relaxation time, as discussed above. We also set *r*_L(R)_ = 1 − *f*_L(R)_ as the probability that singlet or triplet on the left (right) side will be misidentified due to noise. Here *f*_L(R)_ is the measurement fidelity due to random noise on the left (right) pair. The experimentally measured probabilities are

13 $${P}_{{\rm{SS}}}^{\prime}=(1-{r}_{{\rm{L}}}-{r}_{{\rm{R}}}){P}_{{\rm{SS}}}+{g}_{{\rm{L}}}{P}_{{\rm{TS}}}+{g}_{{\rm{L}}}{P}_{{\rm{ST}}}+{r}_{{\rm{L}}}{P}_{{\rm{TS}}}+{r}_{{\rm{R}}}{P}_{{\rm{ST}}},$$

14 $${P}_{{\rm{ST}}}^{\prime}=(1-{r}_{{\rm{L}}}-{r}_{{\rm{R}}}){P}_{{\rm{ST}}}+{g}_{{\rm{L}}}{P}_{{\rm{TT}}}-{g}_{{\rm{L}}}{P}_{{\rm{ST}}}+{r}_{{\rm{L}}}{P}_{{\rm{TT}}}+{r}_{{\rm{R}}}{P}_{{\rm{SS}}},$$

15 $${P}_{{\rm{TS}}}^{\prime}=(1-{r}_{{\rm{L}}}-{r}_{{\rm{R}}}){P}_{{\rm{TS}}}-{g}_{{\rm{L}}}{P}_{{\rm{TS}}}+{g}_{{\rm{L}}}{P}_{{\rm{TT}}}+{r}_{{\rm{L}}}{P}_{{\rm{SS}}}+{r}_{{\rm{R}}}{P}_{{\rm{TT}}},$$

16 $${P}_{{\rm{TT}}}^{\prime}=(1-{r}_{{\rm{L}}}-{r}_{{\rm{R}}}){P}_{{\rm{TT}}}-{g}_{{\rm{L}}}{P}_{{\rm{TT}}}-{g}_{{\rm{L}}}{P}_{{\rm{TT}}}+{r}_{{\rm{L}}}{P}_{{\rm{ST}}}+{r}_{{\rm{R}}}{P}_{{\rm{TS}}}.$$

The displayed probabilities in Fig. 2d are ${P}_{{\rm{SS}}}^{\prime}$, ${P}_{{\rm{ST}}}^{\prime}$, ${P}_{{\rm{TS}}}^{\prime}$, and ${P}_{{\rm{TT}}}^{\prime}$.

To simulate the data shown in Fig. 3, we generate variable exchange propagators *U*_12_ and *U*_34_ using Hamiltonians analogous to Eq. (7) for exchange between qubits 1–2 and qubits 3–4. Probabilities were calculated as described above. For example, to generate the simulations in Supplementary Fig. 3a, the final state is computed as $\left|\psi \right\rangle ={U}_{{\rm{B}}}{U}_{34}{U}_{{\rm{B}}}{S}_{23}{U}_{{\rm{B}}}\left|{\psi }_{i}\right\rangle$. We compute all possible correlators *C*_*α*,*β*_, where *α* is any of $\{\left|S\right\rangle ,\left|{T}_{+}\right\rangle ,\left|{T}_{0}\right\rangle ,\left|{T}_{-}\right\rangle \}$, and *β* is any of $\{\left|\downarrow \uparrow \right\rangle ,\left|{T}_{+}\right\rangle ,\left|\uparrow \downarrow \right\rangle ,\left|{T}_{-}\right\rangle \}$ and extract probabilities as discussed above. The simulated data are averaged over 1000 realizations of magnetic and electrical noise and random state errors. We note that the ground state configuration ($\left|\downarrow \uparrow \right\rangle$ or $\left|\uparrow \downarrow \right\rangle$) is allowed to change in the simulation if the gradient changes sign due to random noise. The results of these simulations are shown in Supplementary Fig. 3, which shows the operator sequences and initial states used to simulate the data.

To simulate the data in Fig. 4, we compute the final state as $\left|\psi \right\rangle ={S}_{12}^{1/2}{U}_{{\rm{B}}}{S}_{23}{U}_{{\rm{B}}}{S}_{12}^{1/2}{U}_{{\rm{B}}}^{{\rm{R}}}(t)\left|{\psi }_{i}\right\rangle$. Here ${U}_{{\rm{B}}}^{{\rm{R}}}(t)$ indicates that the right-pair of qubits evolves for a variable time *t* in their magnetic gradient Δ*B*_34_. We compute all possible correlators *C*_*α*,*β*_, where *α* is any of $\{\left|\downarrow \uparrow \right\rangle ,\left|{T}_{+}\right\rangle ,\left|\uparrow \downarrow \right\rangle ,\left|{T}_{-}\right\rangle \}$, and *β* is any of $\{\left|S\right\rangle ,\left|{T}_{+}\right\rangle ,\left|{T}_{0}\right\rangle ,\left|{T}_{-}\right\rangle \}$. For this simulation, magnetic gradients were chosen to match the observed frequencies, and the width of the hyperfine distribution was reduced to mimic the effects of averaging for only a few seconds and to match the observed decay. For these data, exchange strengths were chosen to be 90 MHz.

To simulate the ideal results in the absence of noise in Fig. 2, Supplementary Figs. 4 and 6, we eliminated all preparation and readout errors, all noise sources, and we eliminated the 2-ns evolution periods, which account for pulse rise times. We also eliminated the effect of magnetic gradients during the SWAP pulses.

### Estimation of Δ*B* Frequencies

To extract the oscillation frequencies of the data in Fig. 4 and Supplementary Fig. 8, we zero-padded each line (corresponding to an average of up to 256 single-shot repetitions of each evolution time) by 256 points and took the absolute value of the fast Fourier transform of this averaged time series. We then found the frequency giving the peak value. To reduce the effects of noise, we rejected all repetitions giving frequencies larger than 100 MHz. To generate the displayed frequency vs. repetition number traces, we smoothed the frequency vs. repetition series with a moving 10-point average.

### Applicability to Si spin qubits

All of the necessary steps for conditional teleportation, including barrier-controlled exchange^23^, and readout and initialization via Pauli spin-blockade^42^, have already been demonstrated in Si quantum dots. In general, we expect teleportation to work even better in Si, where magnetic gradients and noise can be reduced. One potential challenge is the requirement for spin blockade; small valley splittings in Si can easily lift spin-blockade^43^. However, this challenge is easily overcome by operating the quantum dots at larger occupation numbers where the singlet–triplet energy splitting is dominated by the orbital energy spacing^44,45^. Another potential complication for Si qubits is the frequent use of micromagnets, which generate intense magnetic field gradients, for single-spin control. In particular, strong magnetic gradients make pure exchange rotations challenging. However, resonant exchange gates^25,26^ or dynamically corrected gates^27^ can still generate high-fidelity SWAP operations in large magnetic gradients.

### Extension to deterministic teleportation of arbitrary states

Deterministic teleportation of arbitrary states requires the ability to distinguish all four Bell states and the ability to generate arbitrary input states to the teleport. Achieving complete readout in the Bell-state basis is most easily achieved with single-qubit and CNOT gates together with single-qubit readout^32^. High-fidelity single-qubit^31^ and CNOT gates^28–30^ have already been demonstrated in Si. Single-spin readout can be achieved via Pauli spin-blockade measurements with a known ancilla spin^46^, as discussed in the Readout section above, and SWAP operations. Alternatively, spin-selective tunneling^47^ can be used. Fast spin measurements^48^ together with real-time adaptive control^33^ could be used to complete the deterministic state transfer process.

## Supplementary information

Supplementary Information

## Data Availability

The data that support the findings of this study are available from the corresponding author upon reasonable request.
