# Supplementary material for: Conditional teleportation of quantum-dot spin states
Source: Nat Commun. 2020 Jun 15;11:3022. doi: 10.1038/s41467-020-16745-0 (PMC7295761; doi:10.1038/s41467-020-16745-0)
Supplement: Supplementary file 1 — Supplementary Information [file 41467_2020_16745_MOESM1_ESM.pdf]

Supplementary Information -
Conditional Teleportation of Quantum-Dot Spin States

Haifeng Qiao,[1,*] Yadav P. Kandel,[1,*] Sreenath K. Manikandan,[1] Andrew N. Jordan,[1,2] Saeed Fallahi,[3,4] Geoffrey C. Gardner,[4,5] Michael J. Manfra,[3,4,5,6] and John M. Nichol[1,†]

[1]*Department of Physics and Astronomy, University of Rochester, Rochester, NY, 14627 USA*

[2]*Institute for Quantum Studies, Chapman University, Orange, CA 92866, USA*

[3]*Department of Physics and Astronomy, Purdue University, West Lafayette, IN, 47907 USA*

[4]*Birck Nanotechnology Center, Purdue University, West Lafayette, IN, 47907 USA*

[5]*School of Materials Engineering, Purdue University, West Lafayette, IN, 47907 USA*

[6]*School of Electrical and Computer Engineering, Purdue University, West Lafayette, IN, 47907 USA*

SUPPLEMENTARY NOTE 1. ENTANGLEMENT SWAPPING

Here we derive the expected measurement dynamics for the entanglement swapping experiment. We label the quantum dots respectively 1, 2, 3, and 4. The electrons in dots 1 and 2 are initialized in the following two qubit entangled state,

$$|\psi_{12}\rangle = \frac{1}{\sqrt{2}}\Big(e^{i\phi}|\uparrow\rangle_1|\downarrow\rangle_2 - e^{-i\phi}|\downarrow\rangle_1|\uparrow\rangle_2\Big), \tag{1}$$

where we take $\phi = \frac{-\pi}{4}$. The electrons in quantum dots 3 and 4 are also initialized in a two qubit entangled state,

$$|\psi_{34}\rangle = \frac{1}{\sqrt{2}}\Big(e^{i\chi(t)}|\uparrow\rangle_3|\downarrow\rangle_4 - e^{-i\chi(t)}|\downarrow\rangle_3|\uparrow\rangle_4\Big), \tag{2}$$

where $\chi(t)$ is the time dependent phase generated by the magnetic field gradient ΔB_{34}. The joint spin state of four qubits is,

$$\begin{aligned}|\psi_0\rangle &= \frac{1}{\sqrt{2}}\Big(e^{i\phi}|\uparrow\rangle_1|\downarrow\rangle_2 - e^{-i\phi}|\downarrow\rangle_1|\uparrow\rangle_2\Big) \otimes \frac{1}{\sqrt{2}}\Big(e^{i\chi(t)}|\uparrow\rangle_3|\downarrow\rangle_4 - e^{-i\chi(t)}|\downarrow\rangle_3|\uparrow\rangle_4\Big)\\ &= \frac{1}{2}\Big(e^{i[\phi+\chi(t)]}|\uparrow\rangle_1|\downarrow\rangle_2|\uparrow\rangle_3|\downarrow\rangle_4 - e^{-i[\phi-\chi(t)]}|\downarrow\rangle_1|\uparrow\rangle_2|\uparrow\rangle_3|\downarrow\rangle_4\\ &+ e^{-i[\phi+\chi(t)]}|\downarrow\rangle_1|\uparrow\rangle_2|\downarrow\rangle_3|\uparrow\rangle_4 - e^{i[\phi-\chi(t)]}|\uparrow\rangle_1|\downarrow\rangle_2|\downarrow\rangle_3|\uparrow\rangle_4\Big).\end{aligned} \tag{3}$$

In principle, entanglement swapping via joint measurements can be demonstrated on this quantum state, by jointly measuring the qubits 2 and 3 (or 1 and 4), but for the ease of implementing the joint measurement in our four qubit processor, we apply a unitary SWAP operation between the qubits 2 and 3 that swaps the quantum states of electrons between quantum dots 2 and 3, yielding the modified quantum state,

$$\begin{aligned}|\psi_0'\rangle = \frac{1}{2}\Big(&e^{i[\phi+\chi(t)]}|\uparrow\rangle_1|\uparrow\rangle_2|\downarrow\rangle_3|\downarrow\rangle_4 - e^{-i[\phi-\chi(t)]}|\downarrow\rangle_1|\uparrow\rangle_2|\uparrow\rangle_3|\downarrow\rangle_4\\ &+ e^{-i[\phi+\chi(t)]}|\downarrow\rangle_1|\downarrow\rangle_2|\uparrow\rangle_3|\uparrow\rangle_4 - e^{i[\phi-\chi(t)]}|\uparrow\rangle_1|\downarrow\rangle_2|\downarrow\rangle_3|\uparrow\rangle_4\Big).\end{aligned} \tag{4}$$

The electron in quantum dot 1 is now entangled to the electron in quantum dot 3, and the electron in quantum dot 2 is entangled to the electron in quantum dot 4. When we perform a joint measurement of qubits 3 and 4 in the $\{|S\rangle, |T\rangle\}$ basis, and look at cases where the outcome is a singlet, we find that the conditional state of electrons in quantum dots 1 and 2 is,

$$|\Phi_{12}^{S_{34}}\rangle = \langle S_{34}|\psi_0'\rangle = \frac{1}{2\sqrt{2}}\Big(e^{i[\phi-\chi(t)]}|\uparrow\rangle_1|\downarrow\rangle_2 - e^{-i[\phi-\chi(t)]}|\downarrow\rangle_1|\uparrow\rangle_2\Big). \tag{5}$$

Recall $|S\rangle = \frac{1}{\sqrt{2}}\big(|\uparrow\downarrow\rangle - |\downarrow\uparrow\rangle\big)$. We find that, as a result of the joint measurement of the electrons in dots 3 and 4, in cases where the measurement outcome is a singlet entangled state, the reduced state of electrons in dots 1 and 2 is maximally entangled. Also note that the coherent singlet-triplet evolution previously occurring between the qubits 3 and 4 now happens between qubits 1 and 2, provided a singlet is measured on qubits 3 and 4.

We may also derive the unconditional quantum density matrix for qubits 1 and 2, after an ideal joint measurement of qubits 3 and 4 in the $\{S, T\}$ basis. We obtain the following conditional states for qubits 1 and 2 for any of the triplet outcomes on qubits 3 and 4:

$$\begin{aligned}
|\Phi_{12}^{T_{34}^0}\rangle &= \langle T_{34}^0|\psi_0'\rangle = -\frac{1}{2\sqrt{2}}\Big(e^{i[\phi-\chi(t)]}|\uparrow\rangle_1|\downarrow\rangle_2 + e^{-i[\phi-\chi(t)]}|\downarrow\rangle_1|\uparrow\rangle_2\Big),\\
|\Phi_{12}^{T_{34}^+}\rangle &= \langle T_{34}^+|\psi_0'\rangle = \frac{e^{-i[\phi+\chi(t)]}}{2}|\downarrow\rangle_1|\downarrow\rangle_2 = \frac{e^{-i[\phi+\chi(t)]}}{2}\,|T_{12}^-\rangle\\
|\Phi_{12}^{T_{34}^-}\rangle &= \langle T_{34}^-|\psi_0'\rangle = \frac{e^{i[\phi+\chi(t)]}}{2}|\uparrow\rangle_1|\uparrow\rangle_2 = \frac{e^{i[\phi+\chi(t)]}}{2}\,|T_{12}^+\rangle\,. \qquad (6)
\end{aligned}$$

We have denoted $|T^0\rangle = \frac{1}{\sqrt{2}}(|\uparrow\downarrow\rangle + |\downarrow\uparrow\rangle)$, $|T^+\rangle = |\uparrow\uparrow\rangle$ and $|T^-\rangle = |\downarrow\downarrow\rangle$. To simplify notation, in this section we have distinguished the triplet states with superscripts.

It is easily verified that

$$\begin{aligned}
|\Phi_{12}^{S_{34}}\rangle\langle\Phi_{12}^{S_{34}}| + |\Phi_{12}^{T_{34}^0}\rangle\langle\Phi_{12}^{T_{34}^0}| &= \frac{1}{4}\Big(|\uparrow\downarrow\rangle\langle\uparrow\downarrow| + |\downarrow\uparrow\rangle\langle\downarrow\uparrow|\Big)\\
&= \frac{1}{8}\Big(\big(|S\rangle + |T^0\rangle\big)\big(\langle S| + \langle T^0|\big) + \big(|S\rangle - |T^0\rangle\big)\big(\langle S| - \langle T^0|\big)\Big)\\
&= \frac{1}{4}\big(|S\rangle\langle S| + |T^0\rangle\langle T^0|\big)\,. \qquad (7)
\end{aligned}$$

The unconditioned state after measurement in the $\{S, T\}$ basis is therefore,

$$\hat{\rho}_{12} = |\Phi_{12}^{S_{34}}\rangle\langle\Phi_{12}^{S_{34}}| + |\Phi_{12}^{T_{34}^0}\rangle\langle\Phi_{12}^{T_{34}^0}| + |\Phi_{12}^{T_{34}^+}\rangle\langle\Phi_{12}^{T_{34}^+}| + |\Phi_{12}^{T_{34}^-}\rangle\langle\Phi_{12}^{T_{34}^-}| = \frac{\hat{\mathbb{I}}_{4\times4}}{4} \qquad (8)$$

in the $\{S, T\}$ basis. This density matrix represents the completely mixed state, having no time-dependence.

SUPPLEMENTARY NOTE 2. CLASSICAL FIDELITY BOUND FOR ENTANGLEMENT SWAPPING

In this section, we argue that the classical bound on the teleportation fidelity for the entanglement swap experiment is 2/3.

The fidelity of classical teleportation of a single qubit is 2/3 [1]. The reason for this is the following. If Alice wishes to teleport an unknown single qubit quantum state to Bob, she could simply measure her qubit (to the best of her abilities using generalized measurements) and transmit her result to Bob, who could then prepare his qubit according to Alice's measurement. The average probability that Bob's state matches Alice's original state is 2/3. This fidelity estimate is based on the arguments of Ref. [1], and it is the best one can achieve in the absence of systematic errors and not using additional quantum resources. A fidelity exceeding this bound, 2/3 for a single qubit, is a strong indication that additional quantum features are present in the experiment and has been used as a good benchmark to verify successful quantum teleportation.

To extend this result to entangled states of the type we use in this experiment, we note that each pair of qubits initially occupies the $m_s = 0$ subspace, where m_s is the total z-component of angular momentum. Therefore, the state of each pair of qubits is spanned by the $|S\rangle$ and $|T_0\rangle$ joint spin states and is a singlet-triplet qubit. Assuming conservation of m_s, upon post selection of one pair as a singlet, the other pair thus must have $m_s = 0$ and is also a singlet-triplet qubit. Thus, we can view teleportation of entangled states in our experiment as a transfer of singlet-triplet qubit states, and we may ask, how well can Alice classically transmit an individual singlet-triplet qubit. Note also that our experiment involves teleportation not of arbitrary singlet-triplet states, but only states of the form $\cos(\theta/2)\,|S\rangle + \exp(\pm i\pi/2)\sin(\theta/2)\,|T_0\rangle$. Because the fidelity with which Bob can approximate Alice's states does not depend on the azimuthal angle (here $\pm\pi/2$) of her state on her Bloch sphere, we expect that the relevant classical bound for this experiment is identical to the usual single-qubit result of 2/3. Below we provide a proof of this result.

Let $|\psi\rangle = \cos(\theta/2)\,|0\rangle + e^{i\phi}\sin(\theta/2)\,|1\rangle$ be an arbitrary qubit state. Suppose Alice measures $|\psi\rangle$, and finds $|0\rangle$. This occurs with probability $P_0 = \cos^2(\theta/2)$. Bob then prepares his qubit as $|0\rangle$. The fidelity of Bob's qubit with respect to the initial state $|\psi\rangle$ is $S_0 = |\langle 0|\psi\rangle|^2 = \cos^2(\theta/2)$.

Suppose instead that Alice measures $|\psi\rangle$ and finds $|1\rangle$. This occurs with probability $P_1 = \sin^2(\theta/2)$. In this case, the fidelity of Bob's qubit with respect to the initial state is $S_1 = |\langle 1|\psi\rangle|^2 = \sin^2(\theta/2)$.

Define $S(\theta,\phi) = P_0 S_0 + P_1 S_1 = \sin^4(\theta/2) + \cos^4(\theta/2)$ to be the fidelity of Bob's qubit for a particular choice of θ and ϕ. Thus, the average fidelity of Bob's qubit with respect to $|\psi\rangle$,

averaged over all possible states, is

$$F = \frac{\int_0^{2\pi} d\phi \int_0^{\pi} d\theta \sin(\theta) S(\theta,\phi)}{\int_0^{2\pi} d\phi \int_0^{\pi} d\theta \sin(\theta)} = \frac{2}{3}. \tag{9}$$

We now proceed to show that the same bound holds for states $|\psi\rangle$ in the experiment in the $y-z$ plane containing the two poles of the Bloch sphere, $|0\rangle$ and $|1\rangle$. The fidelity computed for this sub-ensemble of states becomes,

$$F' = \frac{\int_0^{2\pi} d\phi \int_0^{\pi} d\theta \sin(\theta) S(\theta,\phi))[\delta(\phi-\pi/2)+\delta(\phi-3\pi/2)]}{\int_0^{2\pi} d\phi \int_0^{\pi} d\theta \sin(\theta)[\delta(\phi-\pi/2)+\delta(\phi-3\pi/2)]} = \frac{2}{3}. \tag{10}$$

The above equation indicates that 2/3 is a classical bound for the fidelity with respect to states in the $y-z$ plane.

Any realistic teleportation experiment involves choosing a subset of all possible input states. A common choice is a complete set of orthogonal input states [2–8]. As we have discussed in the paper, the input states available to us in this work correspond to singlet-triplet qubit states on the $y-z$ plane of the Bloch sphere. When choosing a subset of input states, one must take care not to assume any additional information about the teleportation protocol that could be advantageously used during verification. In the case of F', for example, we do not assume that states following teleportation lie along a particular plane of the Bloch sphere. Full quantum state tomography is required to establish teleportation fidelity above the classical bound. Our assumption that teleportation can take qubit states out of the initial plane justifies the spherical integral discussed above.

SUPPLEMENTARY NOTE 3. ESTIMATED FIDELITY

We simulate the fidelity of the conditional teleport operation for spin eigenstates by starting with the initial state $|\psi_i\rangle = |T_{+,12}\rangle \otimes |\tilde{S}_{34}\rangle$, and we simulate the following operator sequence, as discussed above: $|\psi\rangle = U_B S_{23} U_B |\psi_i\rangle$. We seek to compute the probability that a singlet outcome on the left pair coincides with qubit 4 having the $|\uparrow\rangle$ state. To assess this probability, we compute all correlators $C_{\alpha,\beta}$ as before. To incorporate readout errors and relaxation, we set

$$C'_{|S\rangle,\beta} = (1-r_L)C_{|S\rangle,\beta} + (g_L + r_L)\sum_{\alpha\neq|S\rangle,\beta} C_{\alpha,\beta}. \tag{11}$$

The probability for qubit 4 to have the $|\uparrow\rangle$ state, conditioned on the left pair yielding a singlet, is

$$P_{\uparrow} = \frac{C'_{|S\rangle,|T_+\rangle} + \frac{1}{2}\left(C'_{|S\rangle,|S\rangle} + C'_{|S\rangle,|T_0\rangle}\right)}{\sum_\beta C'_{|S\rangle,\beta}}. \tag{12}$$

We averaged $P_{\uparrow}$ over 100 different realizations of the magnetic and electric noise and state preparation errors, as discussed above.

The fidelity of the combined teleport and SWAP operation for entangled states is determined through a simulation similar to that described above. We initialized the array in the $|\psi_i\rangle = |\tilde{G}_{12}\rangle \otimes |S_{34}\rangle$ state, and the final state is computed as $|\psi\rangle = S_{12}^{1/2} U_B S_{23} U_B S_{12}^{1/2} U_B^R(t) |\psi_i\rangle$. We have assumed perfect state preparation on the right pair of qubits. Now, we compute all possible correlators $C_{\alpha,\beta}$, where α and β are any one of $\{|S\rangle, |T_+\rangle, |T_0\rangle, |T_-\rangle\}$. We incorporate readout errors as above, and the fidelity was computed as the maximum value of $C'_{|S\rangle,|S\rangle}$ as the evolution time t varies in a quasi-static gradient. Choosing the maximum value in this way is equivalent to picking a single-qubit z rotation to undo the effects of singlet-triplet evolution in a gradient.

A first estimate of the fidelity for singlet teleportation may be obtained from our entanglement swap data $p(S_L|S_R)$ by determining the maximum value of $p(S_L|S_R)$ while correcting for left-side readout errors and right-side state preparation errors. To correct the $p(S_L|S_R)$ data for left side readout errors, we invert Eq. 13 to obtain

$$P_{SS} = \frac{P'_{SS} - g_L - r_L}{1 - g_L - 2r_L}. \tag{13}$$

In writing this equation, we have set $r_R = g_R = 0$ and $P_{TS} = 1 - P_{SS}$, since we neglect right-side readout errors and only consider those experimental runs yielding a singlet on the right side. We apply Eq. 13 to our data (P'_{SS}) to extract P_{SS}. We then fit the first period of the resulting data to a function of the form $P(t) = A + B\cos(\omega t + \delta)$, where A, B, ω, and δ are fit parameters. To correct for preparation errors of the state to be teleported, we set $B' = B/(2f_s - 1)$, where f_s is the singlet load fidelity, discussed above. We compute the maximum singlet return probability as $P(15\text{ ns}) = 0.71 \pm 0.04$ with B' used in place of B. The quoted uncertainty is the 95%-confidence interval associated with the fit. The data and fits are shown in Supplementary Fig. 9.

SUPPLEMENTARY NOTE 4. HYPOTHESIS TESTING

We consider the following two hypotheses for the time-series data presented as verification of entanglement swapping,

- Hypothesis Q: Entanglement swapping is achieved using quantum correlations as a resource. This means that the measurement outcome on the left pair of qubits depends on the measurement outcome of the right pair of qubits in a predictable way.
- Hypothesis C: The quantum information is transmitted via classical means. This means that the measurement outcomes on the left pair of qubits would not have a conditional dependence on the measurement outcome on the right pair of qubits.

The key test of quantum effects in this experiment is thus the observation of oscillations on the left pair of qubits that appear only when the left-side measurements are conditioned on the right-side measurements. On the one hand, when entanglement swapping is successful, $p(S_L|S_R)$ and $p(S_L|T_R)$ (shown in Fig. 4(b) in the main text and in Supplementary Fig. 13 here) should both oscillate in time as the input state $|\psi\rangle$ precesses in the $y-z$ plane of the $S-T_0$- qubit Bloch sphere. This oscillation occurs even though the unconditioned data $p(S_L)$ show no oscillations. The absence of oscillations in the unconditioned data happens because on average, Bob's member of the EPR pair and the single-qubit state to be teleported (a member of another entangled pair) are completely uncorrelated, as discussed in detail above.

On the other hand, if the unconditioned data $p(S_L)$ were to show the same oscillation as $p(S_L|S_R)$, for example, the claim of conditional entanglement swapping using quantum resources would be invalidated. This could occur, for instance, when the oscillations on the left pair of qubits are a result of a local preparation/driving when the information about the quantum state on the right pair is received via classical means (i.e. classical teleportation).

As discussed in the caption to Supplementary Fig. 6, the amplitude of oscillations associated with $p(S_L|T_R)$ is expected to be smaller than the oscillations associated with $p(S_L|S_R)$ because our measurements do not distinguish the three triplets, which are linear combinations of the three Bell states $|\Psi^+\rangle$, $|\Phi^+\rangle$, and $|\Phi^-\rangle$.

To assess the probability of the classical hypothesis (C), we first quantify the amplitude of the residual oscillations in the unconditioned data $p(S_L)$, which may occur because of incomplete averaging, or an imperfect SWAP. We fit these data to a function of the form

$g(t) = p_0 + Ae^{-t/\tau}\cos(2\pi ft + \phi)$, where p_0, A, τ, ω, and ϕ are fit parameters. The unconditioned data have an amplitude $A_U = 0.030 \pm 0.007$. Here the uncertainty is a standard error σ_U. Similarly, to test the quantum hypothesis (Q), we fit the data $p(S_L|S_R)$ and $p(S_L|T_R)$ to the same function, and we extract $A_S = 0.201 \pm 0.021$ and $A_T = 0.102 \pm 0.009$. The uncertainties are the standard errors σ_S and σ_T, respectively. Our fitting routine in Matlab computes the confidence intervals using the t-distribution. Using the t-distribution, we convert the confidence interval to a standard error. Table 1 shows the fitted parameters, standard errors σ, and 95% confidence intervals c. Note the close agreement between the usual relation $c = 1.96\sigma$ for a normal distribution. Note that $\sigma_U < \sigma_S \ll A_S - A_U$, and $\sigma_U < \sigma_T \ll A_T - A_U$, suggesting a very low probability for the classical hypothesis.

Define

$$G(x, \sigma, \mu) = \frac{1}{\sqrt{2\pi}\sigma} e^{-\frac{1}{2}\left(\frac{x-\mu}{\sigma}\right)^2}. \tag{14}$$

The probability of the classical hypothesis for the case of singlet conditioning is the probability that the amplitude of $p(S_L|S_R)$ is less than A_U. Assuming that the amplitude values of $p(S_L|S_R)$ are normally distributed between experimental runs, we assess this probability as

$$p_S = \int_{-\infty}^{A_U} G\left(x, \sigma_S, A_S\right) dx < 0.00001. \tag{15}$$

Note that since $\sigma_S > \sigma_U$, it is reasonable to neglect the variance of A_U to a first approximation. The probability of a classical explanation for the case of triplet conditioning is the probability that the amplitude of $p(S_L|T_R)$ is less than A_U. We assess this probability as

$$p_T = \int_{-\infty}^{A_U} G\left(x, \sigma_T, A_T\right) dx < 0.00001. \tag{16}$$

Since $\sigma_T < \sigma_U$, it is also reasonable to neglect the variance of A_C to a first approximation.

To account for the variance of A_U, we may perform Welch's t-test accounting for unequal sample variances,

$$t_S = \frac{A_S - A_U}{\sqrt{\sigma_U^2 + \sigma_S^2}} = 7.72, \tag{17}$$

and

$$t_T = \frac{A_T - A_U}{\sqrt{\sigma_U^2 + \sigma_T^2}} = 6.32, \tag{18}$$

We use the Welch–Satterthwaite equation to estimate the number of degrees of freedom, finding 150 and 232 for the cases of singlet and triplet conditioning respectively. The p-values associated with the t_S and t_T scores both satisfy $p < 0.00001$. Given these probabilities, a classical explanation for the data is extremely unlikely.

A further, more conservative estimate for the classical hypothesis probability is obtained by analyzing all of the data shown in Supplementary Fig. 7. For each repetition $i = 1, 2, \cdots, 256$, we calculate $U_i = \frac{\max(p(S_L)) - \min(p(S_L))}{2}$, $S_i = \frac{\max(p(S_L|S_R)) - \min(p(S_L|S_R))}{2}$, and $T_i = \frac{\max(p(S_L|T_R)) - \min(p(S_L|T_R))}{2}$. S_i, T_i, and U_i quantify the maximum amplitude of the singlet-conditioned, triplet-conditioned, and unconditioned time series data for each repetition i.

Supplementary Figure 14 shows the distributions of S_i, T_i, and U_i over all 256 different repetitions. We calculate $\mu_S = 0.234$, $\sigma_S = 0.036$, $\mu_T = 0.162$, $\sigma_T = 0.021$, $\mu_U = 0.113$, and $\sigma_U = 0.015$. Here, μ indicates an average, and σ refers to the estimated standard deviation. As before, we compute the t-scores associated with these distributions:

$$t_S = \frac{\mu_S - \mu_U}{\sqrt{\sigma_U^2/N + \sigma_S^2/N}} = 49.46, \tag{19}$$

and

$$t_T = \frac{\mu_T - \mu_U}{\sqrt{\sigma_U^2/N + \sigma_T^2/N}} = 29.92, \tag{20}$$

where $N = 256$ is the sample size. These large t-score values result from the large sample sizes and well-separated distributions. Using the Welch–Satterthwaite equation, we find the number of degrees of freedom to be 339 and 463 for singlet and triplet conditioning. The p-values associated with these t-scores both satisfy $p < 0.00001$. Both p-values are below 0.05, indicating that the classical hypothesis is extremely unlikely.

We may assess the impact of potential spurious classical correlations in our data as follows. As discussed in the main text, the data shown in Supplementary Fig. 5 represent the same experiment as the entanglement swap, but the SWAP gate to distribute the entangled pair was omitted. Thus, the left-side measurements in Supplementary Fig. 5(a) provide a bound on the presence of oscillations caused by classical means, including readout cross-talk. We have fit these data as described above in this section, and as shown in Supplementary Fig. 15. The fitted parameters are shown in Supplementary Table 2.

We may determine if the observed oscillations in the control experiment are statistically different from the oscillations associated with the full entanglement-swap data using the

following t scores:

$$t_S^c = \frac{A_S - A_S^c}{\sqrt{\sigma_S^2 + (\sigma_S^c)^2}} = 7.01, \tag{21}$$

and

$$t_T^c = \frac{A_T - A_T^c}{\sqrt{\sigma_T^2 + (\sigma_T^c)^2}} = 3.05, \tag{22}$$

Here, the variables with a "c" superscript are obtained from Supplementary Table 2. In both of these cases, we obtain the degrees of freedom as discussed above, and the p-values associated with the t scores both satisfy $p < 0.005$. Given these probabilities, it is unlikely that classical correlations could explain our data.

	$p(S_L)$			$p(S_L\|S_R)$			$p(S_L\|T_R)$		
Parameter	value	c	σ	value	c	σ	value	c	σ
p_0	0.362	0.007	0.004	0.464	0.011	0.006	0.287	0.008	0.004
A	0.030	0.014	0.007	0.201	0.042	0.021	0.102	0.017	0.009
f (GHz)	0.026	.002	0.001	0.028	0.0005	0.0003	0.029	0.0008	0.0004
ϕ	4.54	0.76	0.38	3.84	0.19	0.10	0.557	0.271	0.137
τ (ns)	151	0	0	142	71	36	151	0	0

Supplementary Table 1. Fitted parameters. c denotes half of the 95%-confidence interval width, and σ is the standard error. In the case of the unconditioned data $p(S_L)$ and $p(S_L|T_R)$, we did not fit for the decay time τ. Instead, we fixed it at the value obtained from the fit to $p(S_L|S_R)$.

	$p(S_L)$			$p(S_L\|S_R)$			$p(S_L\|T_R)$		
Parameter	value	c	σ	value	c	σ	value	c	σ
p_0	0.534	0.006	0.003	0.538	0.008	0.004	0.521	0.010	0.005
A	0.010	0.015	0.008	0.038	0.019	0.010	0.056	0.024	0.012
f (GHz)	0.027	0	0	0.027	0	0	0.027	0	0
ϕ	4.46	1.51	0.763	-0.92	0.54	0.27	2.38	0.47	0.24
τ (ns)	78	0	0	78	0	0	78	0	0

Supplementary Table 2. Fitted parameters for the control experiment. c denotes half of the 95%-confidence interval width, and σ is the standard error. We used the data in Supplementary Fig. 5(b) to fix the values of f and τ in the fits.

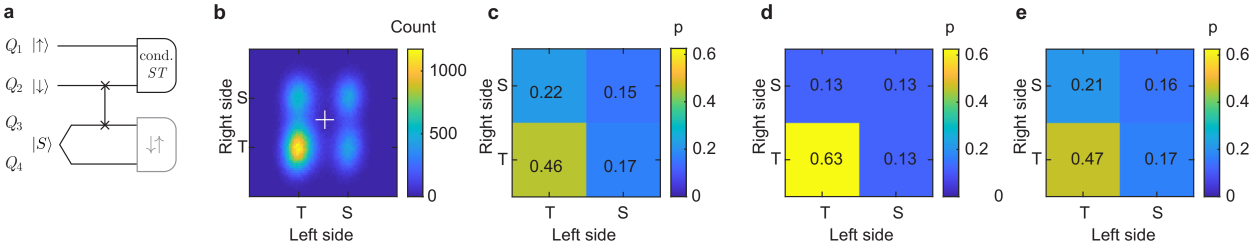

Supplementary Figure 1. Conditional teleportation of a classical mixed spin state. (a) Quantum circuit used. The conditional singlet-triplet measurement on qubits 1 and 2 induces teleportation, and the gray measurement of the right pair verifies teleportation. In contrast to the experiment of Fig. 2, in this experiment, we adiabatically separate the electrons in the left pair. This process generates either $|\downarrow\rangle_1 |\uparrow\rangle_2$ or $|\uparrow\rangle_1 |\downarrow\rangle_2$, depending on the sign of the hyperfine gradient ΔB_{12}. The hyperfine gradient fluctuates randomly during the course of the experiment, leading to the generation of a classical mixed state on qubit 1 (and qubit 2). Likewise, the measurement on the right pair projects either $|\downarrow\rangle_3 |\uparrow\rangle_4$ or $|\uparrow\rangle_3 |\downarrow\rangle_4$ to $|S_{34}\rangle$, depending on the sign of ΔB_{34}. (b) Experimentally measured probability distribution for 65,536 single-shot realizations of the teleportation sequence in (a). The white cross indicates the threshold used to calculate probabilities. (c) Extracted probabilities p from the distribution in (b). (d) Simulated probabilities computed neglecting any errors. (e) Simulated probabilities accounting for readout errors, state preparation errors, charge noise, and hyperfine fields. All probabilities are rounded to the nearest hundredth.

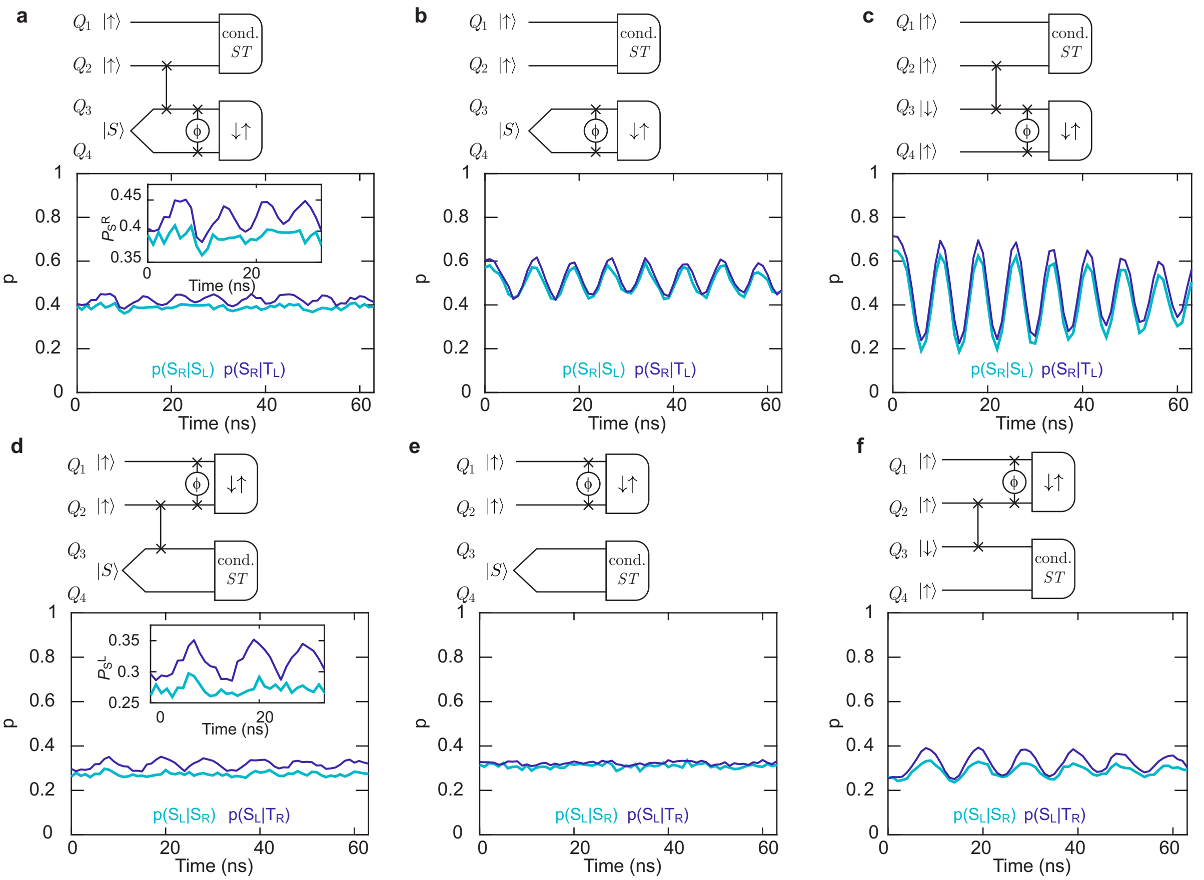

Supplementary Figure 2. Verification of conditional teleportation of a classical spin state. (a) We apply a variable exchange gate to the right pair after measuring the left pair. Here, $\phi = 2\pi J_{34} t$, where J_{34} is the exchange coupling between qubits 3 and 4, and t is the evolution time given by the x-coordinate of each data point. ($\phi = \pi$ corresponds to a SWAP operation.) When then left pair give a singlet, the right pair have the same spin, and no oscillations should be visible. The inset shows the same data from 0-32 ns. (b) Control experiment with no SWAP operation. Residual oscillations result from errors in preparing the EPR pair. (c) Control experiment with the EPR pair replaced by a product state. Oscillations occur because the fluctuating hyperfine gradient between the right pair sometimes favors the $|\uparrow\downarrow\rangle$ orientation. (d) Applying an exchange gate to the left pair after measuring the right pair generates exchange oscillations on the left pair only if the right pair yields a triplet. The inset shows the same data from 0-32 ns. (e) Control experiment with no SWAP operation. No oscillations occur because qubits 1 and 2 are prepared in the $|\uparrow\uparrow\rangle$ state. (f) Control experiment with the EPR pair replaced by a product state. Here, the oscillations are small because the hyperfine gradient between qubits 3 and 4 fluctuates around zero. Each data point represents the average of 16,384 single shot measurements.

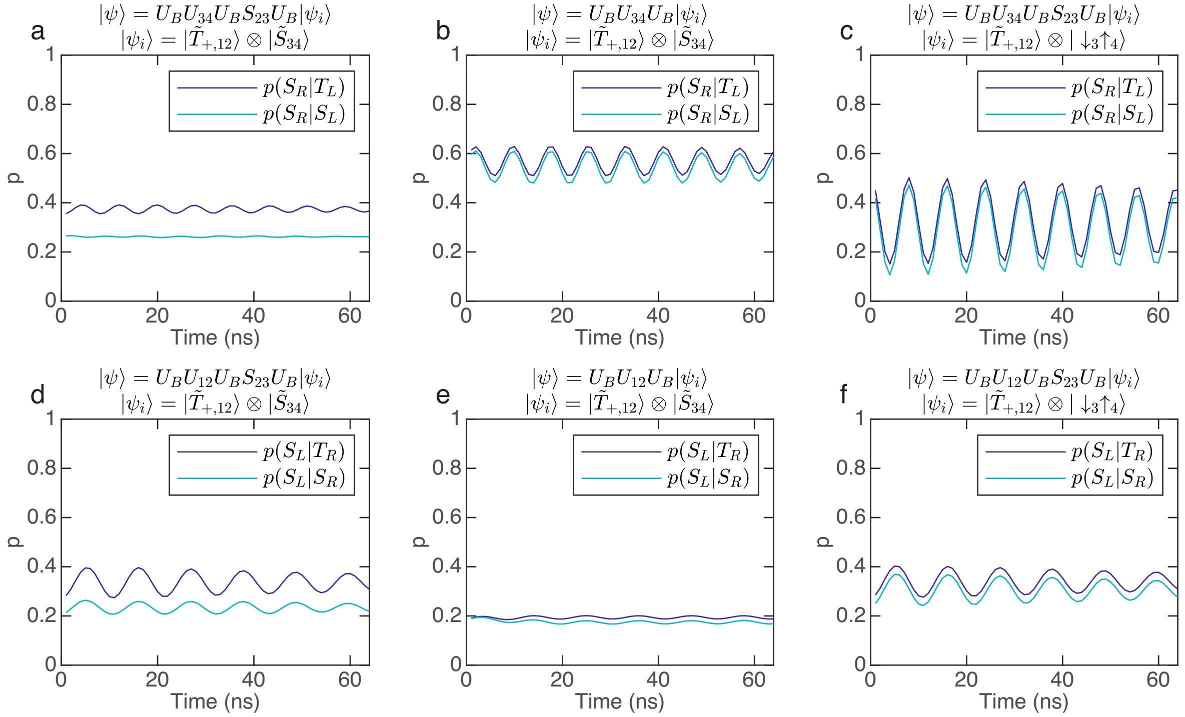

Supplementary Figure 3. Simulated variable-exchange experiments. Panels (a)-(f) present a simulation of the corresponding panel in Supplementary Fig. 2. Above each panel is the operator sequence used in the simulation and the initial state used in the simulation. In panels (a)-(c), $p(S_R|S_L)$ indicates the right-side singlet probability given a singlet on the left, and $p(S_R|T_L)$ indicates the right-side singlet probability given a triplet on the left. In (d)-(f), $p(S_L|S_R)$ indicates the left-side singlet probability given a singlet on the right, and $p(S_L|T_R)$ indicates the left-side singlet probability given a triplet on the right.

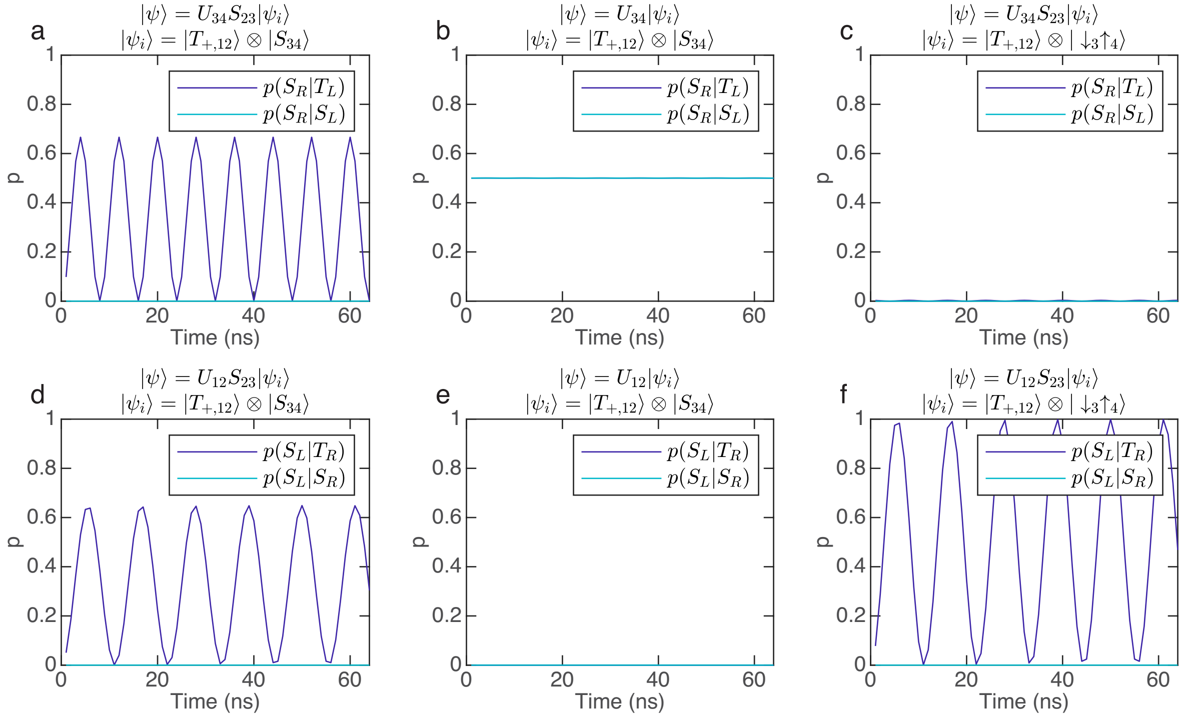

Supplementary Figure 4. Simulated variable-exchange experiments with no errors associated with pulses, hyperfine fluctuations, state preparation, or readout. Panels (a)-(f) present a simulation of the corresponding panel in Supplementary Fig. 2. In panels (a)-(c), $p(S_R|S_L)$ indicates the right-side singlet probability given a singlet on the left, and $p(S_R|T_L)$ indicates the right-side singlet probability given a triplet on the left. In (d)-(f), $p(S_L|S_R)$ indicates the left-side singlet probability given a singlet on the right, and $p(S_L|T_R)$ indicates the left-side singlet probability given a triplet on the right. The oscillations in (a) and (b) have visibility 2/3, as expected. In the case of (a), for example, there is a 1/3 chance that a triplet outcome on the left corresponds to a $|T_0\rangle$, in which case successful teleportation of the $|\uparrow\rangle$ state occurs. The dominant mechanism that reduces the visibility of these oscillations in practice is the fluctuating sign of the hyperfine gradient. The absence of oscillations in (c) is a result of the fixed hyperfine gradient in this simulation. For example, if the gradient were such that the ground state of the right pair were $|\uparrow_3\rangle\,|\downarrow_4\rangle$, oscillations with unit visibility would be expected. Similarly, the unit-visibility oscillations in panel (f) occur because of the assumed ground orientation of qubits 3 and 4. The apparent conditional effect in this panel occurs because there is zero probability to measure a singlet on the right side, i.e., $p(S_R) = 0$.

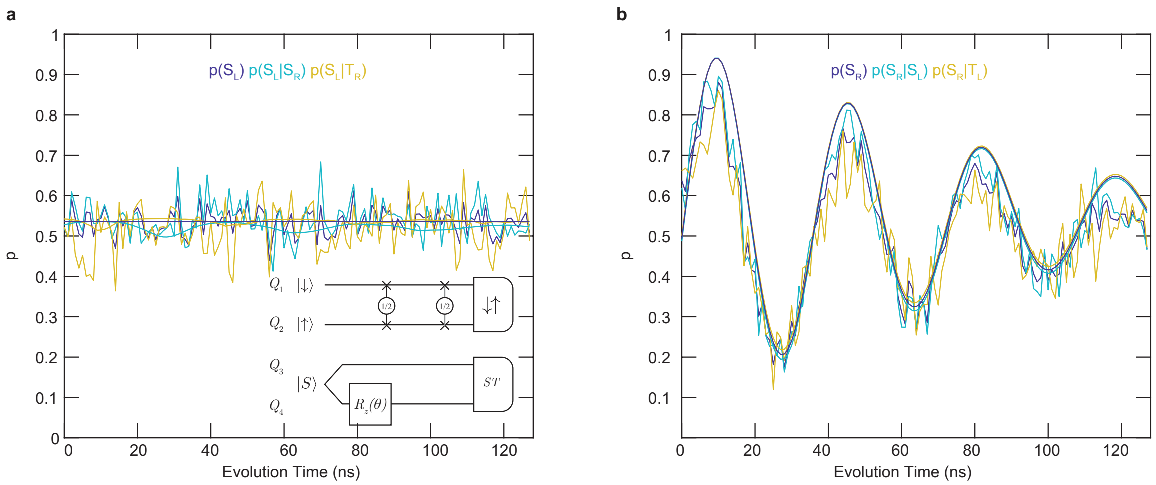

Supplementary Figure 5. Control experiments for entanglement swapping and gate teleportation without the SWAP operation between qubits 2 and 3. To omit the SWAP gate, we enforce a wait for the same length of time with no voltage pulse. (a) Measured singlet probabilities on the left side. As before, simulations are overlaid in smooth lines of the same color. Inset: circuit diagram for the control experiment. (b) Measured right-side probabilities. Simulations are overlaid with smooth lines of the same color. The absence of any conditional effect in the left- and right-side measurements confirms that the behavior we observe in Fig. 4 is due to entanglement swapping and gate teleportation. These data were acquired in a separate run from the data in Fig. 4 in the main text. The data in panels (a) and (b) of this figure are from one repetition.

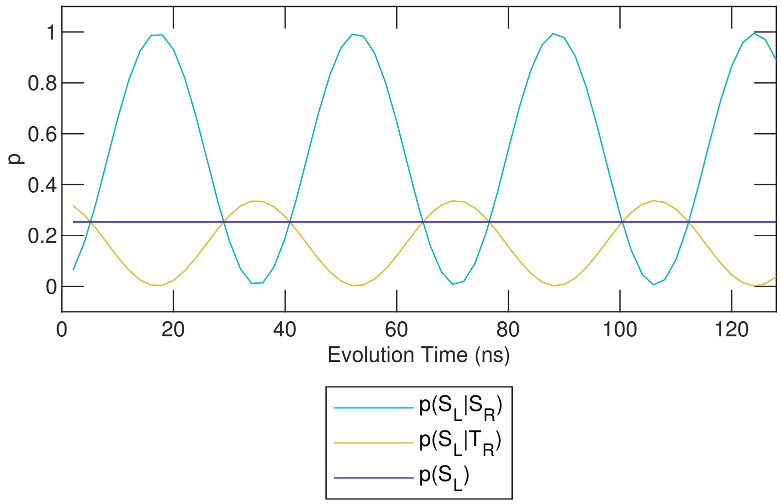

Supplementary Figure 6. Simulated entanglement swapping with no errors associated with pulses, hyperfine fluctuations, state preparation, or readout. The oscillations associated with $p(S_L|T_R)$ occur with visibility 1/3, because in 1/3 of the cases where we measure a triplet on the right pair, it is a $|T_0\rangle$, and successful teleportation has occurred.

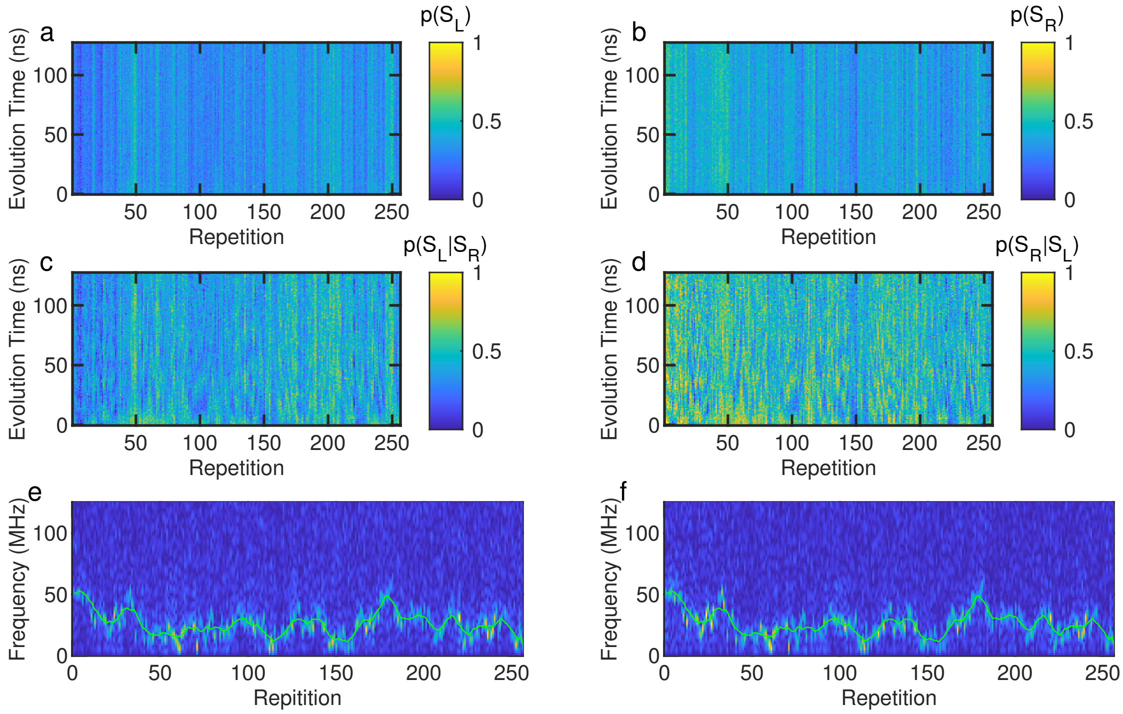

Supplementary Figure 7. Conditional teleportation of entangled states and conditional gate teleportation. (a) Averaged singlet probability on the left pair of qubits $p(S_L)$. (b) Averaged singlet probability on the right pair of qubits $p(S_R)$. (c) Averaged singlet probability on the left pair of qubits given a singlet on the right pair $p(S_L|S_R)$. (d) Averaged singlet probability on the right pair of qubits given a singlet on the left pair $p(S_R|S_L)$. (e) Absolute value of the fast Fourier transform of the data in (c). The extracted peak frequency is overlaid in green. (f) Absolute value of the fast Fourier transform of the data in (d). The extracted peak frequency is overlaid in green.

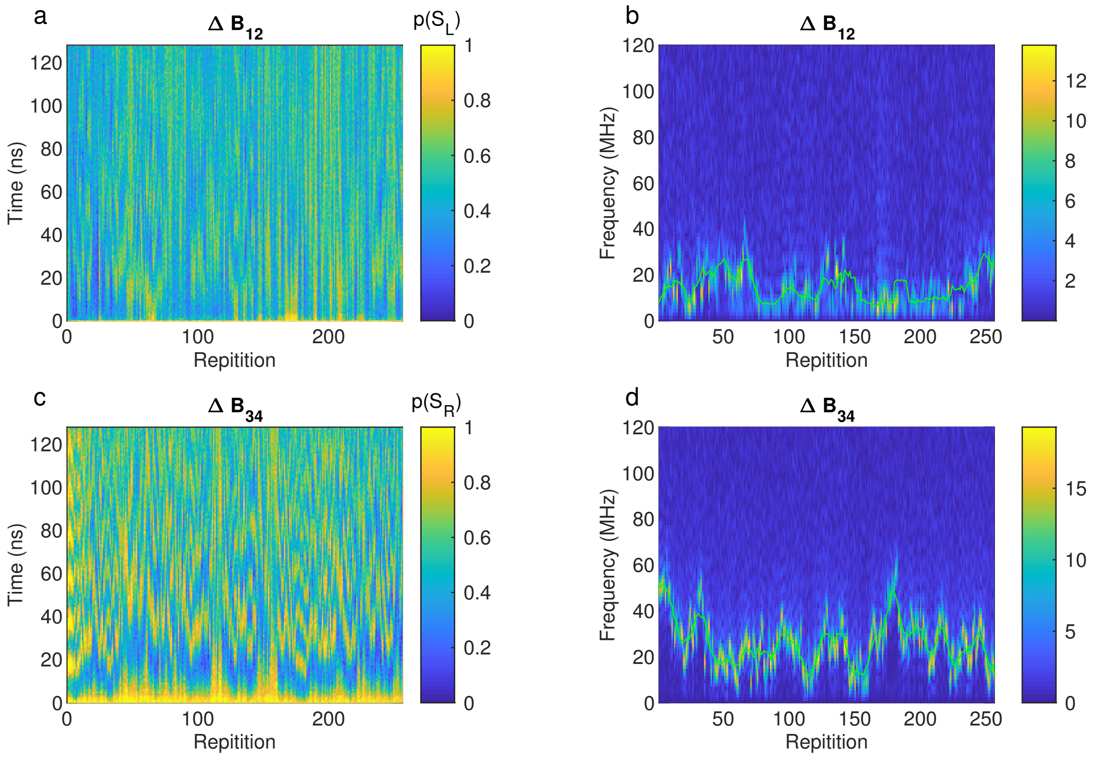

Supplementary Figure 8. Measurement of ΔB. (a) Averaged measurements of ΔB_{12} oscillations [9]. Acquisition of each vertical line was interleaved with the data shown in Fig. 4. (b) Absolute value of the fast Fourier transform of the data in (a), with the extracted frequency shown in green. (c) Averaged measurements of ΔB_{34} oscillations. Acquisition of these data were also interleaved with the data in Fig. 4. (d) Absolute value of the fast Fourier transform of the data in (c), with the extracted frequency shown in green.

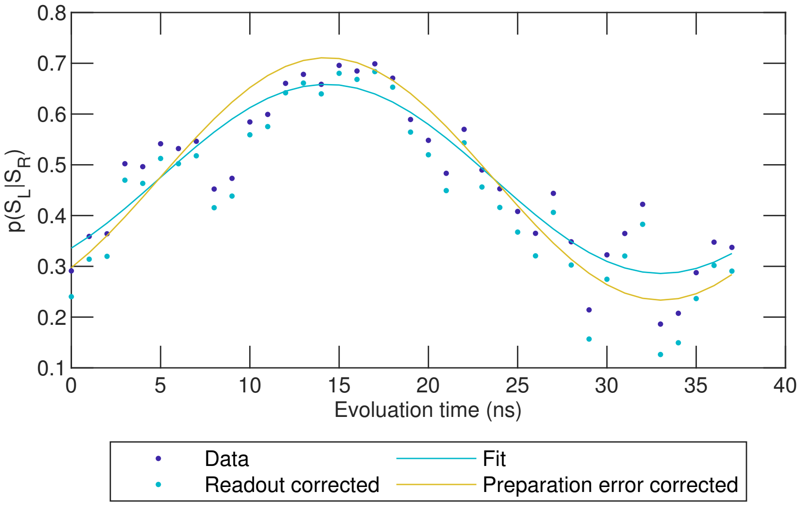

Supplementary Figure 9. Experimental estimation of the maximum singlet teleportation probability. The dark blue dots are the data $p(S_L|S_R)$ from Fig. 4(b). The light blue dots have been corrected for left-side readout errors. The light-blue line is a fit of the light-blue dots to a sinusoid. The gold line is the same sinusoid with the amplitude corrected for the right-side preparation error. The maximum singlet return probability is obtained at about 15 ns.

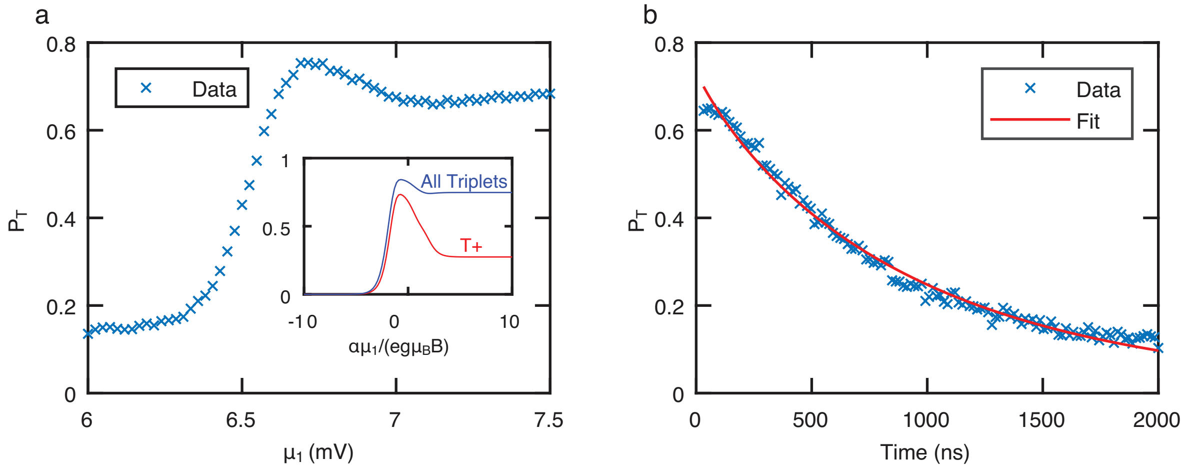

Supplementary Figure 10. State preparation fidelity. (a) Experimentally measured $|T_+\rangle$ loading curve, obtained by sweeping μ_1, the electrochemical potential of dot 1, across the (1,1)-(2,1) charge transition, where (m,n) indicates m electrons in dot 1 and n electrons in dot 2 [10, 11]. The peak in the data indicates where the value of μ_1 where the $|T_+\rangle$ loading probability is highest. Inset: Simulated results of the loading process. The blue simulation gives the expected triplet signal, and the red simulation is the probability of loading a $|T_+\rangle$. The simulation assumes a load time of 2 μs, a ramp time of 200 ns, a temperature of 75 mK, and a magnetic field of 0.5 T. The x axis represents the chemical potential $\alpha\mu_1$ in units of the magnetic field, where α is the effective lever-arm. (b) Triplet probability as a function of loading time. All experiments were conducted with a loading time of 2 μs. The blue points are data, and the red line is a fit to an exponential decay. These data are taken in the same tuning used to acquire all data in this work with one electron in each dot.

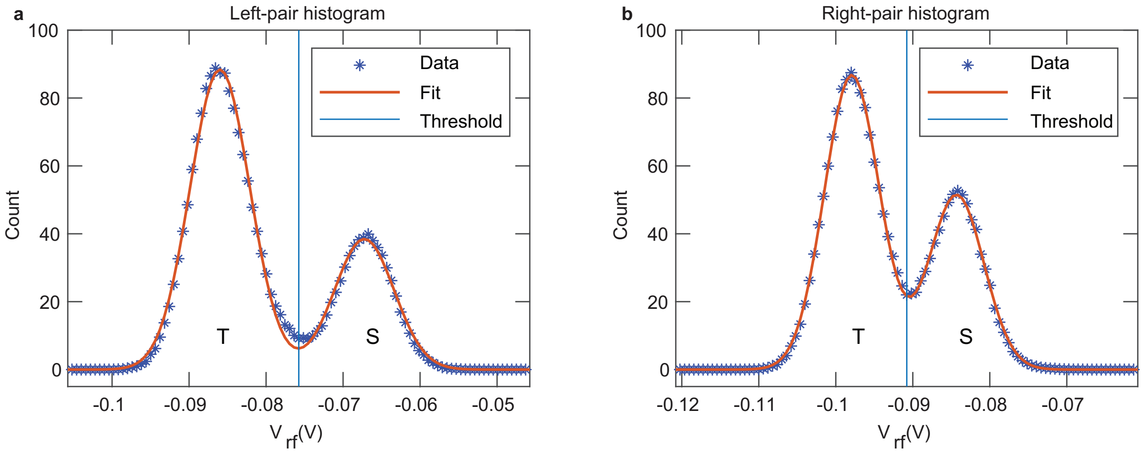

Supplementary Figure 11. Readout fidelity. (a) Measurement histogram and fit for the left pair of qubits for the data shown in Fig. 2(b). The extracted average fidelity for singlets and triplets is 0.93. (b) Measurement histogram and fit for the right pair of qubits for the data shown in Fig. 2(a). The extracted average fidelity for both singlets and triplets is 0.87. In both panels, the red lines are fits to equations (1) and (2) of Ref. [12]. In both panels, the V_{RF} represents the raw voltage from our readout circuit. In both panels, the threshold which maximizes the visibility is indicated.

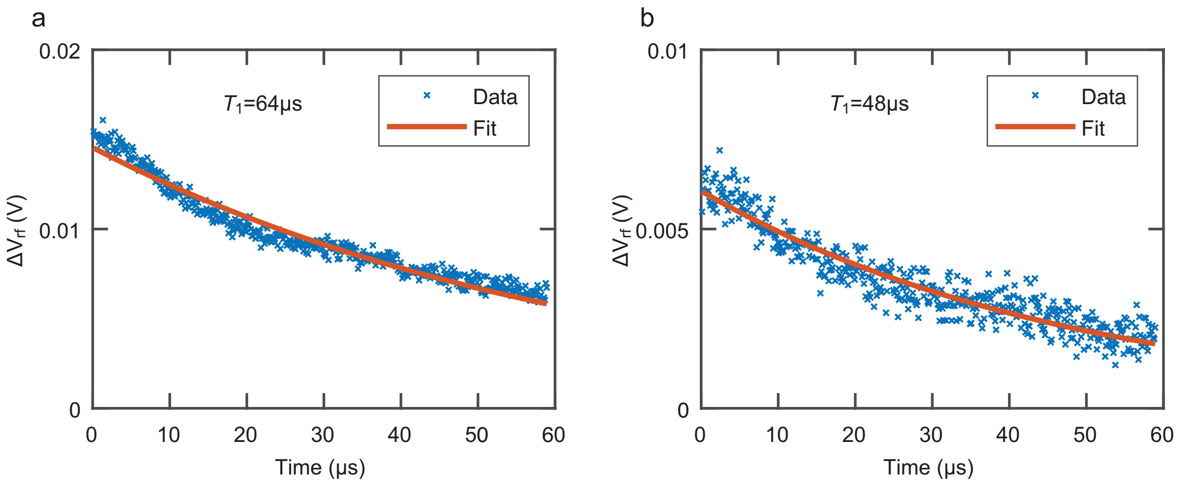

Supplementary Figure 12. Relaxation during readout. (a) Data and fit showing the relaxation of the left pair of qubits during readout. (b) Data and relaxation of the right pair of qubits during readout. The data in both panels are obtained by subtracting the results of two measurements. The first involves preparing a mixed state and measuring it for 60 μs. Then, we prepare a singlet state and also measure it for 60 μs. We repeat this set of two measurements 10,000 times. For each repetition, we record the entire 60 μs measurement record. We plot the difference of the two experiments, averaged across all repetitions, as a function of integration time from 0-60 μs. We fit this difference to a decaying exponential and extract the relaxation time T_1. The fits give relaxation times of 65 μs and 48 μs. These data are taken in the same tuning used to acquire all data in this work with one electron in each dot. In both panels, there appears to be a slight deviation from single-exponential decay. We attribute this deviation to the multiple relaxation pathways and rates involved in our shelving readout method. [10, 13].

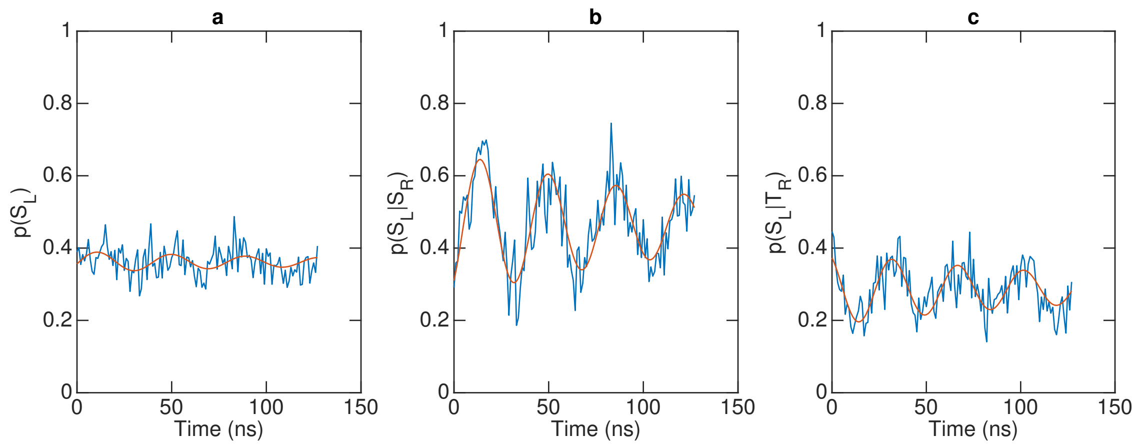

Supplementary Figure 13. Data and fits. (a) $p(S_L)$ from Fig. 4b of the main text and fit. (b) $p(S_L|S_R)$ from Fig. 4b of the main text and fit. (c) $p(S_L|T_R)$ from Fig. 4b of the main text and fit. These data and fits are used to assess the probability that a classical teleportation strategy could reproduce the results of the entanglement-swap experiment.

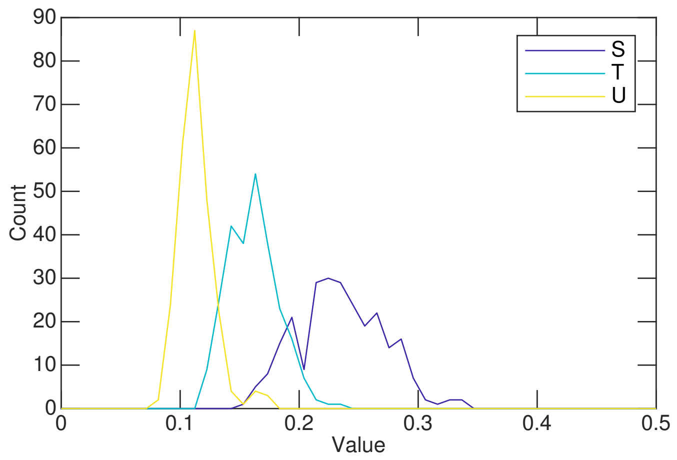

Supplementary Figure 14. Distributions of S_i, T_i, and U_i. The pronounced difference between these distributions indicates that a classical explanation for the entanglement-swap experiment is unlikely.

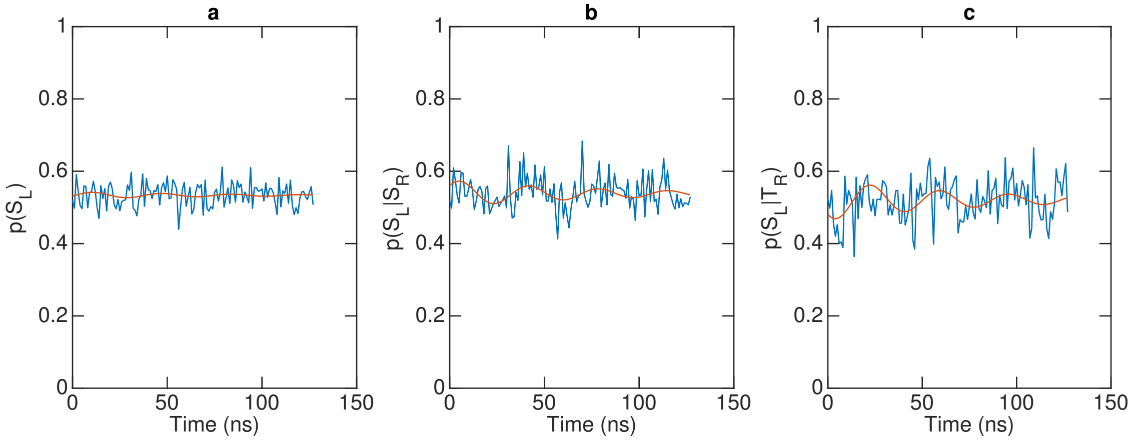

Supplementary Figure 15. Data and fits for the control experiment. (a) $p(S_L)$ from Supplementary Fig. 5 and fit. (b) $p(S_L|S_R)$ from Supplementary Fig. 5. and fit. (c) $p(S_L|T_R)$ from Supplementary Fig. 5. and fit. These data and fits are used to assess the probability that spurious classical correlations could explain the entanglement-swap data.

SUPPLEMENTARY REFERENCES

* These authors contributed equally.

† john.nichol@ur.rochester.edu

[1] S. Massar and S. Popescu, "Optimal extraction of information from finite quantum ensembles," Phys. Rev. Lett. **74**, 1259–1263 (1995).

[2] Dik Bouwmeester, Jian-Wei Pan, Klaus Mattle, Manfred Eibl, Harald Weinfurter, and Anton Zeilinger, "Experimental quantum teleportation," Nature **390**, 575–579 (1997).

[3] M. Riebe, H. Häffner, C. F. Roos, W. Hänsel, J. Benhelm, G. P. T. Lancaster, T. W. Körber, C. Becher, F. Schmidt-Kaler, D. F. V. James, and R. Blatt, "Deterministic quantum teleportation with atoms," Nature **429**, 734–737 (2004).

[4] S. Olmschenk, D. N. Matsukevich, P. Maunz, D. Hayes, L.-M. Duan, and C. Monroe, "Quantum teleportation between distant matter qubits," Science **323**, 486–489 (2009).

[5] L. Steffen, Y. Salathe, M. Oppliger, P. Kurpiers, M. Baur, C. Lang, C. Eichler, G. Puebla-Hellmann, A. Fedorov, and A. Wallraff, "Deterministic quantum teleportation with feed-forward in a solid state system," Nature **500**, 319 (2013).

[6] W. Pfaff, B. J. Hensen, H. Bernien, S. B. van Dam, M. S. Blok, T. H. Taminiau, M. J. Tiggelman, R. N. Schouten, M. Markham, D. J. Twitchen, and R. Hanson, "Unconditional quantum teleportation between distant solid-state quantum bits," Science **345**, 532–535 (2014).

[7] S. Pirandola, J. Eisert, C. Weedbrook, A. Furusawa, and S. L. Braunstein, "Advances in quantum teleportation," Nature Photonics **9**, 641 (2015).

[8] Marcus Reindl, Daniel Huber, Christian Schimpf, Saimon F. Covre da Silva, Michele B. Rota, Huiying Huang, Val Zwiller, Klaus D. Jöns, Armando Rastelli, and Rinaldo Trotta, "All-photonic quantum teleportation using on-demand solid-state quantum emitters," Science Advances **4** (2018), 10.1126/sciadv.aau1255.

[9] M. D. Shulman, S. P. Harvey, J. M. Nichol, S. D. Bartlett, A. C. Doherty, V. Umansky, and A. Yacoby, "Suppressing qubit dephasing using real-time Hamiltonian estimation," Nature Communications **5**, 5156 (2014).

[10] Lucas A. Orona, John M. Nichol, Shannon P. Harvey, Charlotte G. L. Bøttcher, Saeed Fallahi,

Geoffrey C. Gardner, Michael J. Manfra, and Amir Yacoby, "Readout of singlet-triplet qubits at large magnetic field gradients," Phys. Rev. B **98**, 125404 (2018).

[11] Tim Botzem, Michael D. Shulman, Sandra Foletti, Shannon P. Harvey, Oliver E. Dial, Patrick Bethke, Pascal Cerfontaine, Robert P. G. McNeil, Diana Mahalu, Vladimir Umansky, Arne Ludwig, Andreas Wieck, Dieter Schuh, Dominique Bougeard, Amir Yacoby, and Hendrik Bluhm, "Tuning methods for semiconductor spin qubits," Phys. Rev. Applied **10**, 054026 (2018).

[12] C. Barthel, D. J. Reilly, C. M. Marcus, M. P. Hanson, and A. C. Gossard, "Rapid Single-Shot Measurement of a Singlet-Triplet Qubit," Physical Review Letters **103**, 160503 (2009).

[13] S. A. Studenikin, J. Thorgrimson, G. C. Aers, A. Kam, P. Zawadzki, Z. R. Wasilewski, A. Bogan, and A. S. Sachrajda, "Enhanced charge detection of spin qubit readout via an intermediate state," Applied Physics Letters **101**, 233101 (2012).
